# Modeling trajectories of routine blood tests as dynamic biomarkers for outcome in spinal cord injury

**DOI:** 10.1038/s41746-025-01782-0

**Published:** 2025-07-22

**Authors:** Marzieh Mussavi Rizi, Daniel Fernández, John L. K. Kramer, Rajiv Saigal, Anthony M. DiGiorgio, Michael S. Beattie, Adam R. Ferguson, Nikos Kyritsis, Abel Torres-Espín, TRACK-SCI, Prakruthi Amar Kumar, Michael S. Beattie, Jacqueline C. Bresnahan, Anthony M. DiGiorgio, Xuan Duong-Fernandez, Adam R. Ferguson, Debra D. Hemmerle, J. Russel Huie, Anastasia Keller, Nikos Kyritsis, Nicole Lai, Geoffrey T. Manley, Jonathan Z. Pan, Lisa U. Pascual, Rajiv Saigal, Vineeta Singh, Jason F. Talbott, Abel Torres-Espin, Phil Weinstein, William D. Whetstone

**Affiliations:** 1 School of Public Health Sciences, University of Waterloo, Waterloo, ON Canada; 2 Department of Statistics and Operations Research (DEIO), Universitat Politècnica de Catalunya BarcelonaTech (UPC), Barcelona, Spain; 3 International Collaboration on Repair Discoveries (ICORD), University of British Columbia, Vancouver, BC Canada; 4 Department of Anesthesiology, Pharmacology, and Therapeutics, Faculty of Medicine, University of British Columbia, Vancouver, BC Canada; 5 Weill Institute for Neuroscience, Brain and Spinal Injury Center (BASIC), University of California San Francisco, San Francisco, CA USA; 6 Department of Neurological Surgery, University of California San Francisco, San Francisco, CA USA; 7 Zuckerberg San Francisco General Hospital and Trauma Center, University of California, San Francisco, CA USA; 8 San Francisco Veterans Affairs Healthcare System, San Francisco, CA USA; 9 Department of Physical Therapy, Faculty of Rehabilitation Medicine, University of Alberta, Edmonton, AB Canada; 10 Department of Anesthesia and Perioperative Care, University of California San Francisco, San Francisco, CA USA; 11 Orthopaedic Trauma Institute, Department of Orthopedic Surgery, University of California, San Francisco, CA USA; 12 Department of Neurology, University of California San Francisco, San Francisco, CA USA; 13 Department of Radiology and Biomedical Imaging, University of California San Francisco, San Francisco, CA USA; 14 Department of Emergency Medicine, University of California San Francisco, San Francisco, CA USA

**Keywords:** Health care, Biomarkers, Predictive markers, Neurology, Neurological disorders, Medical research, Biomarkers, Statistics

## Abstract

Routinely collected blood tests can reflect underlying pathophysiological processes. We demonstrate that the dynamics of routinely collected blood tests hold prediction validity in acute Spinal Cord Injury (SCI). Using MIMIC data (*n* = 2615) for modeling and TRACK-SCI study data (*n* = 137) for validation, we identified multiple trajectories for common blood markers. We developed machine learning models for the dynamic prediction of in-hospital mortality, SCI occurrence in spine trauma patients, and SCI severity (motor complete vs. incomplete). The in-hospital mortality model achieved an out-of-train ROC-AUC of 0.79 [0.77–0.81] day one post-injury, improving to 0.89 [0.88–0.89] by day 21. For detecting the presence of SCI after spine trauma, the highest ROC-AUC was 0.71 [0.69–0.72] achieved by day 21. By day seven, the ROC-AUC for SCI severity was 0.81 [0.77–0.85]. Our full models outperformed the severity score SAPS II following seven days of hospitalization.

## Introduction

Rare injuries such as traumatic spinal cord injury (SCI), which often require intensive care, present unique challenges in early decision-making that could benefit from the use of routinely collected clinical information and real-world data. Traumatic SCI is a global health challenge, with an estimated 930,000 new cases annually and a prevalence of 20.6 million worldwide in 2019, causing significant personal and economic burden^1–3^. The complexity of SCI, characterized by variable clinical presentations and recovery trajectories, complicates acute diagnosis and prognosis, particularly in emergency departments and intensive care units (ICU)^4–9^. Early neurological assessment of SCI is the main tool for characterizing the injury; however, it is limited by dependence on patient responsiveness and the presence of comorbid injuries^10^. Objective measures such as MRI^11,12^, physiological time series^13–15^, and fluid omics-based biomarkers^16–22^ are being investigated, though their accessibility across medical settings may be limited^6^.

There is growing interest in utilizing routinely collected in-hospital data, such as blood laboratory values, as biomarkers in SCI^15,16^ and other neurological conditions such as Alzheimer’s^23,24^. Spinal cord damage triggers pathophysiological events measurable in the blood^20,22^, and hematologic abnormalities following SCI correlate with injury severity and neurological outcome^22,23^. However, the correlation between a specific marker and critical aspects of the injury—such as its severity, location, and the patient’s neurological recovery—is poor^15,16,21^. Moreover, any marker may be influenced by factors other than SCI, including demographics, concomitant injuries, and other comorbidities. The unpredictable and non-linear progression of SCI and irregular timing of routine in-hospital data collection further complicate their application.

We hypothesized that routine blood biomarkers and their changes over time provide valuable, predictive information for SCI-related outcomes and that incorporating new data over time improves prediction performance. Capturing the evolution of blood biomarkers during a patient’s hospital stay and their relationship with SCI over time represents a novel perspective that has not been widely explored. To test this hypothesis, we propose machine learning models to predict SCI outcomes using longitudinal data from routine blood tests in the early stages of ICU stay, leveraging data from two sources: the electronic health records (EHR) of the Medical Information Mart for Intensive Care (MIMIC)^25,26^ and the Transforming Research and Clinical Knowledge in Spinal Cord Injury (TRACK-SCI)^21,27^ study.

**Fig. 1:**
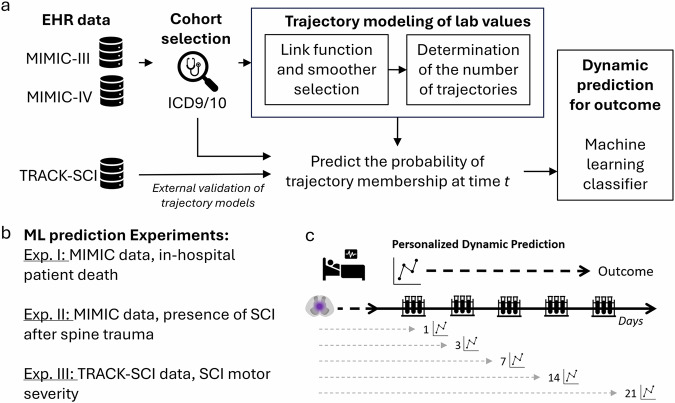
Summary of the multi-stage analysis and computational experiments. **a** Flowchart of the multi-stage analysis. After cohort selection from MIMIC data using ICD9 and 10 codes, lab values were curated and processed for modeling. The trajectory modeling for each marker is performed by first doing a model search to select the best link function and degrees of freedom for the smoother function (natural cubic spline), followed by a linear search of the number of trajectory classes. The selected final models are used for predicting the probability of trajectory membership per subject at different timepoints from hospitalization. These probabilities are then used as predictors in ML classifiers. **b** Three prediction experiments were set up to study the potential use of blood trajectories as dynamic biomarkers. **c** Schematic of the dynamic prediction experiments simulating increased data availability over time after hospital arrival.

Our approach captures the multidimensional, nonlinear, and temporal changes in multiple blood markers over time, rather than relying on single measurements at static time points. We applied a multi-stage modeling (Fig. 1), by first modeling blood biomarker trajectories using longitudinal, finite mixture models, identifying distinct patient subgroups with unique pathophysiological profiles. The probability of membership of these trajectory-based subgroups was then used as dynamic predictors in machine learning classifiers to predict in-hospital mortality, the presence of SCI after spine trauma, and SCI severity. Our findings demonstrate that longitudinal changes in blood lab values can serve as effective dynamic biomarkers in SCI. In practice, this approach could provide real-time predictions based on information available at each time point, enabling earlier identification of SCI patients at higher risk of adverse outcomes, allowing clinicians to intervene proactively.

## Results

### Patient cohort for routine blood trajectory modeling

**Fig. 2:**
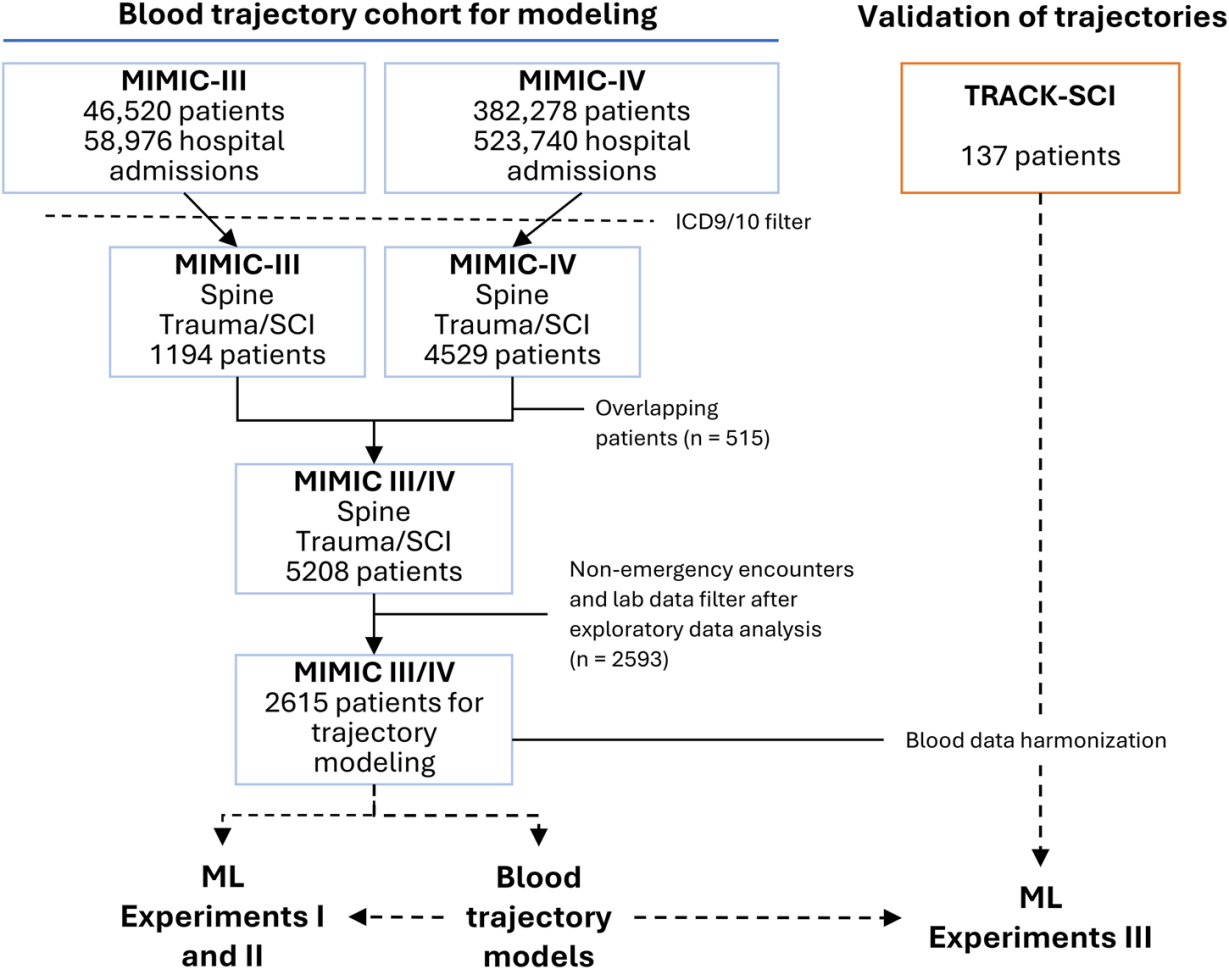
Flow diagram of cohort build. Patients from MIMIC-III/IV were first filtered based on their ICD9 and ICD10 diagnostic codes. Then, potential overlapping patients were filtered from MIMIC-IV. After laboratory analyte data extraction and data cleaning, patients with less than 3 measures for any of the 20 most common analytes were excluded. The data from a total of 2615 patients from both MIMIC databases were used for trajectory modeling as well as Experiments I and II. An additional 137 patients from the TRACK-SCI dataset were used as a validation set in Experiment III to predict SCI severity based on AIS grade.

A total of 1194 and 4529 unique patients were identified from MIMIC-III and MIMIC-IV, respectively. After filtering, a final cohort of 2615 patients with processed laboratory marker data were included for modeling (Fig. 2).

Table 1 shows demographic variables for the selected cohorts from MIMIC datasets (SCI Fracture, SCI noFracture, and Spine Trauma). There were statistical differences in age, gender, insurance type, ethnicity, and dataset (i.e., MIMIC III or IV) across the three groups. The SCI Fracture cohort was younger and had higher proportions of males than the Spine Trauma cohort. A higher proportion of patients in the SCI Fracture group came from the MIMIC-III database (60%), while in the SCI noFracture and Spine Trauma groups, ~60% of patients came from the MIMIC-IV database. Table 2 shows the hospital stay characteristics for MIMIC and the length of stay distribution is shown in Supplementary Fig. 1. We observed statistical differences in length of stay and discharge location. The SCI Fracture patients stayed in hospital a median of 3 and 4 days longer than SCI noFracture and the Spine Trauma groups, respectively. The SCI Fracture group had a higher mortality rate (12%), and a higher proportion of patients that were discharged to rehabilitation (54%) than the other two groups. On the other side, SCI Fracture group were less likely to be discharged home (15%) than the SCI noFracture (37%) and the Spine Trauma (34%) groups.

**Table 1:** Demographics for the MIMIC cohorts

Characteristic	SCI Fracture N = 382^a^	SCI noFracture N = 125^a^	Spine Trauma N = 2108^a^	*p*-value^2^	*q*-value^3^
Age	55 (39, 71)	57 (44, 72)	65 (44, 81)	<0.001	<0.001
Males	281 (74%)	88 (70%)	1184 (56%)	<0.001	<0.001
Insurance				0.011	0.011
Medicaid	37 (9.7%)	13 (10%)	154 (7.3%)		
Medicare	118 (31%)	48 (38%)	865 (41%)		
Other	221 (58%)	62 (50%)	1064 (50%)		
Other Government	6 (1.6%)	2 (1.6%)	25 (1.2%)		
Ethnicity				<0.001	<0.001
ASIAN	6 (1.9%)	0 (0%)	43 (2.3%)		
BLACK/AFRICAN AMERICAN	14 (4.4%)	23 (20%)	88 (4.8%)		
HISPANIC/LATINO	14 (4.4%)	9 (7.9%)	77 (4.2%)		
MULTI RACE/ETHNICITY	1 (0.3%)	0 (0%)	5 (0.3%)		
OTHER	17 (5.4%)	5 (4.4%)	84 (4.6%)		
WHITE	264 (84%)	77 (68%)	1540 (84%)		
Unknown	66	11	271		
Dataset				<0.001	<0.001
MIMIC-III	231 (60%)	49 (39%)	826 (39%)		
MIMIC-IV	151 (40%)	76 (61%)	1282 (61%)		

^a^Median (IQR); *n* (%).

^b^Kruskal–Wallis rank sum test; Fisher’s Exact Test for Count Data with simulated *p*-value (based on 2000 replicates).

^c^False discovery rate correction for multiple testing.

**Table 2:** Hospital stay characteristics for the MIMIC cohorts

Characteristic	SCI Fracture N = 382^a^	SCI noFracture N = 125^a^	Spine Trauma N = 2108^a^	*p*-value^b^	*q*-value^c^
Length of stay (days)	11 (7, 19)	8 (5, 13)	7 (4, 11)	<0.001	<0.001
Unknown	1	0	0		
Admission location				0.079	0.10
CLINIC REFERRAL	55 (14%)	22 (18%)	251 (12%)		
EMERGENCY ROOM	301 (79%)	89 (71%)	1730 (82%)		
TRANSFER FROM HOSP	20 (5.3%)	12 (9.6%)	95 (4.5%)		
TRANSFER FROM SNF	0 (0%)	0 (0%)	3 (0.1%)		
WALK-IN/SELF REFERRAL	4 (1.1%)	2 (1.6%)	20 (1.0%)		
Unknown	2	0	9		
Discharge location				<0.001	<0.001
ACUTE HOSPITAL	4 (1.0%)	0 (0%)	9 (0.4%)		
AGAINST ADVICE	0 (0%)	2 (1.6%)	11 (0.5%)		
DIED	46 (12%)	6 (4.9%)	141 (6.9%)		
HOME	54 (15%)	46 (37%)	687 (34%)		
HOSPICE	2 (0.5%)	1 (0.6%)	22 (0.8%)		
ICF	1 (0.3%)	0 (0%)	0 (0%)		
LONG TERM CARE	22 (5.8%)	5 (4.1%)	88 (4.3%)		
REHAB	208 (54%)	45 (37%)	528 (26%)		
SHORT TERM CARE	3 (0.8%)	0 (0%)	8 (0.4%)		
SKILLED NURSING FACILITY	35 (9.2%)	17 (14%)	544 (27%)		
TRANSFER TO OTHER	7 (1.8%)	2 (1.6%)	29 (1.4%)		
Unknown	0	2	63		
Number of ICD diagnostics	13 (9, 18)	13 (8, 21)	14 (9, 20)	0.10	0.10

^a^Median (IQR); *n* (%).

^b^Kruskal–Wallis rank sum test; Fisher’s Exact Test for Count Data with simulated *p*-value (based on 2000 replicates).

^c^False discovery rate correction for multiple testing.

Supplementary Table 1 shows demographic differences between both MIMIC datasets. Except for ethnicity, we observed substantial differences between the MIMIC cohorts. MIMIC-IV individuals were older (median 70 vs. 53 years old) and higher proportion of individuals had Meidcare insurance (46% vs. 30%). The proportion of males and females was also different, with MIMIC-III showing a 2:1 ratio male:female (67% male, 33% female), and MIMIC-IV an approximately 1:1 ratio (54% male, 46% female). All measured hospital stay characteristics were significantly different between the two MIMIC datasets (Supplementary Table 2). MIMIC-III individuals spent three more days in hospital (median: 9 vs. 6), were more likely to have been admitted from clinical referral (21% vs. 6.2%), and considerably more likely to be discharged to a rehabilitation facility (42% vs. 22%). On the other hand, MIMIC-IV individuals were more likely to be discharged to a skilled nursing facility (33% vs. 10%) and present five more ICD diagnostic codes (median 16 vs. 11). TRACK-SCI characteristics are shown in Supplementary Table 3.

**Fig. 3:**
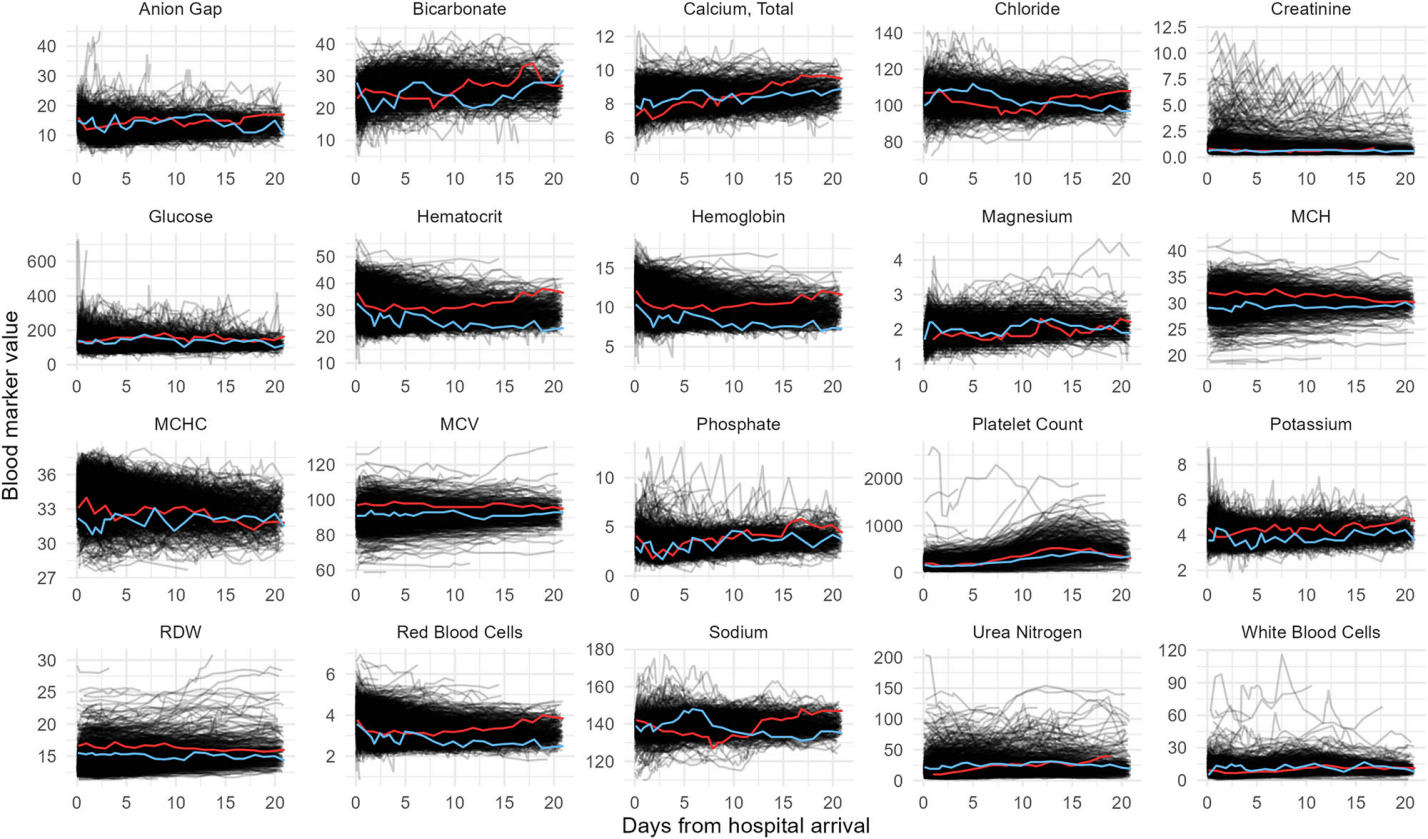
Spaghetti plots for the outlier-cleaned modeling set of laboratory analytes for the first 21 days after admission in the MIMIC data. Each line represents a single subject. Lines red and blue are two randomly selected subjects illustrating differences in temporal trends.

A total of 413 distinct laboratory tests were identified from the MIMIC dataset across blood gases, chemistry, and hematology (Supplementary Table 4). The 20 most common markers across subjects were selected for trajectory modeling (see methods). The spaghetti plots of the marker modeling set over time from the date of hospital arrival for MIMIC patients are shown in Supplementary Fig. 2, with the corresponding plots after outlier filtering presented in Fig. 3. The spaghetti plots for the outlier-cleaned TRACK-SCI modeling set of laboratory analytes over the first 21 days post-admission are provided in Supplementary Fig. 3.

### Routine blood trajectory models

Blood trajectories for the 20 most common markers for up to 21 days from hospital admission were modeled using longitudinal finite mixture models^28^. To explore their use as dynamic biomarkers, we then set three dynamic prediction modeling experiments, using the latent trajectory memberships of each marker as predictors in a machine learning classifier (Fig. 1).

**Fig. 4:**
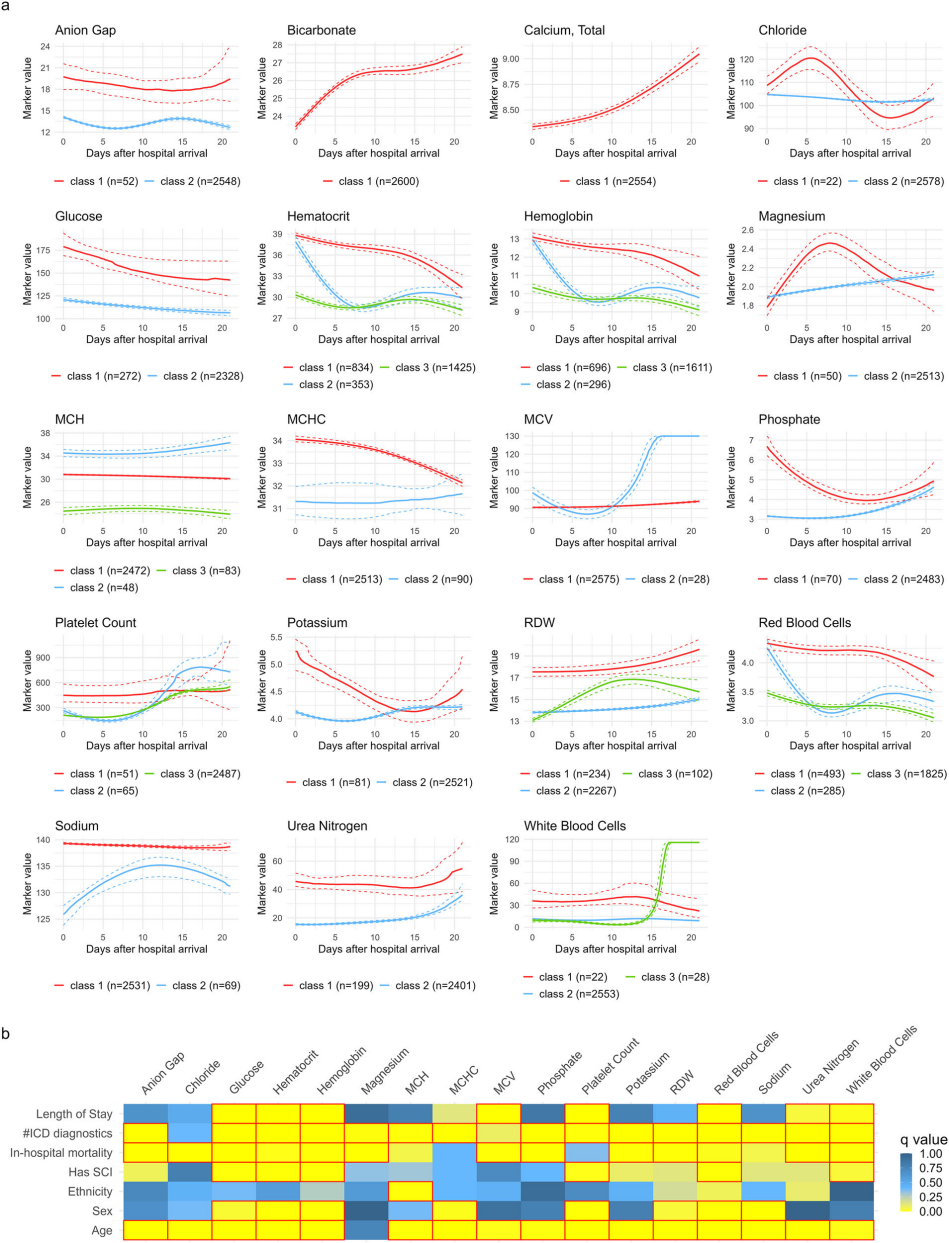
Blood trajectories from MIMIC data. **a** The mean trajectory for each one the classes for each analyte model are shown, together with the 95% CI. Note that although colored the same for visualization, these are univariate models and therefore classes might not be constituted by the same subjects across analytes. The legend in each panel shows the number of subjects assigned to each trajectory class. **b** Univariate summary of association of the different trajectory classes with demographics and other variables of interest. The data are shown as a heatmap of the *q* value (adjusted *p* value for multiple comparisons). Red boxes indicate a *q* value < 0.05.

Figure 4a shows the predicted trajectories for the final selected models of each blood marker, as presented in Supplementary Table 5. Creatinine models did not converge and were excluded. Except for bicarbonate and calcium, models with more than one class were selected as the best fit. Anion gap, chloride, creatinine, magnesium, MCH, MCV, phosphate, platelet count, potassium, sodium, urea nitrogen, and white blood cells selected models had one dominant class containing above 90% of the subjects, often with a small secondary class. Biomarkers glucose, hematocrit, hemoglobin, MCHC, RDW, and red blood cells presented a more balanced distribution of subjects across classes. All markers except glucose were better modeled with non-linear time transformations, and 11 of 20 blood values were best modeled with non-Gaussian link functions. The average APPA across 25 repeats per marker, timepoint, and class was between 0.6 and 0.99 for the modeling (MIMIC, Supplementary Fig. 4) and the validation (TRACK-SCI, Supplementary Fig. 5) data, indicating an overall high certainty in assigning subjects to a class.

Supplementary Fig. 6 provides an illustrative example of final model selection and decision-making using model criteria plots for red blood cell count and potassium models. For red blood cell count, the polynomial degree is the dominant determinant of better model fit by both BIC and ICL. In these cases, we conclude that non-linear trends are a better fit and that the type of link function is almost irrelevant. This suggests that assuming a Gaussian response is reasonable for markers where the link function has minimal impact on model selection. For the selection of the number of trajectory classes regarding red blood cell count, BIC decreases progressively as the number of classes increases, while ICL shows a more pronounced drop at two classes but remains relatively stable thereafter. APPA decreases more gradually with increasing classes. Given that APPA reached acceptable levels in most cases, we used BIC and ICL as the main decision-making tools for this initial step, leaving APPA as a secondary factor. We concluded then that a linear link and a polynomial of order 3 are best for this variable, selecting between 2 and 4 trajectory classes for modeling red blood cell count. The final choice of 3 classes was guided by the balance between model fit (BIC, ICL) and classification stability (APPA), with preference for a more parsimonious model if fit improvements were marginal. While there is still a noticeable improvement on model fit moving from 2 to 3 classes, the improvement is minimal from 3 to 4 classes in ICL. Given the higher cost in number of model parameters to estimate from a 4-cluster model (30 parameters) vs. the 3-cluster model (25 parameters), with little gain in performance, the 3-cluster model is chosen given its parsimony.

In a different example looking at potassium, both the polynomial order and the shape of the link function significantly impact model fit. In this case, models with polynomials of order 3, a link function of type beta or spline, and 2 or 3 trajectory classes were selected for further exploration. This selection reflects the need for a flexible response function, suggesting that assuming a Gaussian response would be inappropriate for potassium.

In summary, most markers were best represented by models with at least two trajectory classes, with a median of three, as supported by model selection criteria. This suggests heterogeneity in the selected cohort. Additionally, most models demonstrated improved fit with polynomial transformations of degree 2 or 3, suggesting that non-linear trends enhance trajectory representation in these data. Finally, with the exception of bicarbonate, chloride, hematocrit, hemoglobin, MCHC, and red blood cell count, models with non-linear link functions (beta or I-spline) provided a superior fit, suggesting that flexible transformations better capture the relationship between predictors and responses for these markers.

Univariate analysis comparing class trajectories across demographics and clinical characteristics for each marker is summarized in Fig. 4b, with detailed results in Supplementary Tables 6–14. Most blood biomarkers showed significant differences in age, in-hospital mortality rates, and number of diagnostics across trajectory classes. Among chemical markers, only glucose and sodium exhibited differences in gender distribution between trajectory classes. For hematology markers, class 1 cases of hematocrit, hemoglobin, and red blood cell counts showed higher initial values followed by a decline, associated with lower mortality rates and fewer diagnostics compared to classes 2 and 3 for each marker. Length of stay and cohort group distribution varied across classes for glucose, hematocrit, hemoglobin, platelet count, red blood cell counts, and white blood cell counts (Supplementary Tables 6–14).

### Blood trajectories as dynamic biomarkers for SCI diagnosis and prognosis

We provide a summary description of the model performance for the ROC-AUC out-of-train sample (mean [95%CI] over 25 independent experimental repeats). ROC-AUC over the training sample, as well as PR-AUC metrics, can be found in the supplementary material (Supplementary Figs. 7–9, Supplementary Tables 15–17), providing a complete evaluation of our models. These models were benchmarked by comparing their performance to models using only SAPS II scores as predictors. Since SAPS II scores were limited to ICU patients, the sample was restricted to ICU admissions in MIMIC. Compared to our models from Experiment I and Experiment II, models incorporating trajectories, summary statistics, and baseline covariates outperformed those using SAPS II alone (Supplementary Figs. 10–13, Supplementary Tables 18–21).

**Fig. 5:**
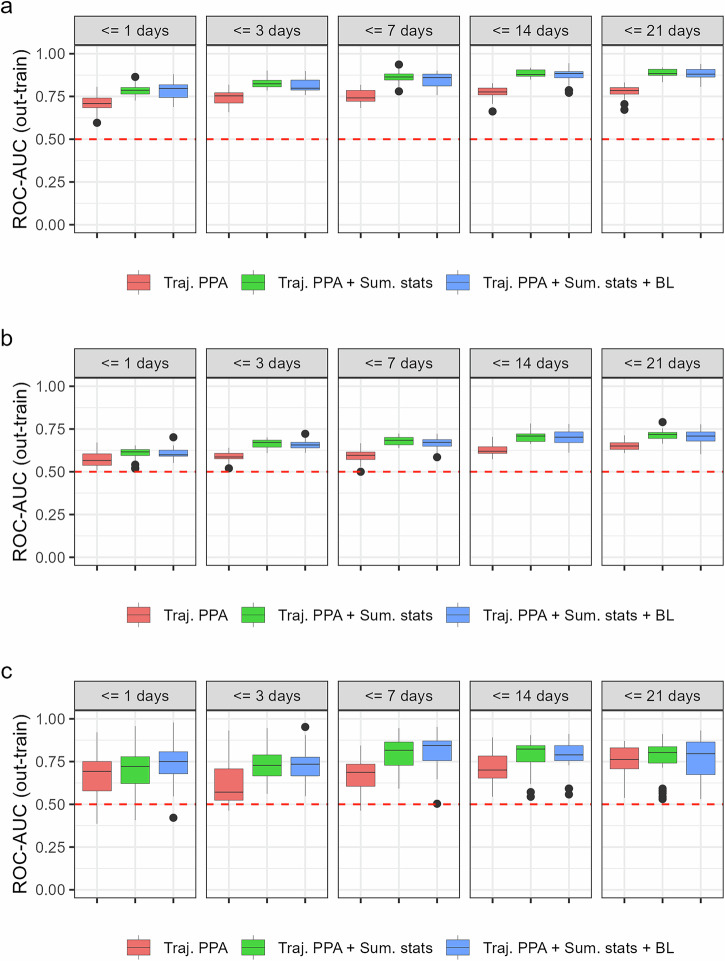
Out-train ROC-AUC performance of dynamic predictions. **a** ROC-AUC out-of-train sample performance of experiment I in-hospital mortality. **b** ROC-AUC out-of-train sample performance of experiment II for detecting the presence of SCI after spine trauma. **c** ROC-AUC out-of-train sample performance of experiment III on detecting SCI severity on the TRACK-SCI cohort, external to trajectory modeling. Dashed red lines represent the non-information rate (mean prevalence of the outcome of interest in each experiment). Three predictors’ lists are shown: Traj. PPA = posterior probability of class assignment only; + Sum. stats = addition of summary statistics of blood data; and + BL = addition of baseline predictors.

For Experiment I, predicting mortality, models trained using only the trajectory features exhibited moderate to high ROC-AUC values for predictions made within the out-of-train sample across all time points (day 1: 0.71 [0.69–0.73] to day 21: 0.78 [0.76–0.79]) (Table 3). Model performance improved over time and with the inclusion of summary statistics and baseline predictors, with the highest ROC-AUC reached at 21 days for the models including trajectory and summary statistics predictors(0.89 [0.88–0.89]) (Table 3, Fig. 5a). SAPS II alone had a ROC-AUC of 0.85 [0.84–0.86], and the addition of SAPS II to the complete list of predictors generated the model with the highest ROC-AUC (at 14 days, 0.89 [0.87–0.9]) (Supplementary Fig. 11).

**Table 3:** ROC AUC performance for out-of-train sample per experiment and feature set over the different time cutoffs

Exp.	Feature set	ROC AUC < = 1 day	ROC AUC < = 3 days	ROC AUC < = 7 days	ROC AUC < = 14 days	ROC AUC < = 21 days
Exp. I	Traj. PPA	0.71 [0.69-0.73]	0.74 [0.72-0.76]	0.75 [0.73-0.77]	0.77 [0.76-0.79]	0.78 [0.76-0.79]
	Traj. PPA + Sum. stats	0.79 [0.77-0.80]	0.83 [0.82-0.84]	0.86 [0.85-0.88]	0.88 [0.87-0.89]	0.89 [0.88-0.90]
	Traj. PPA + Sum. stats + BL	0.79 [0.77-0.81]	0.82 [0.80-0.83]	0.85 [0.83-0.86]	0.87 [0.85-0.89]	0.88 [0.87-0.89]
Exp. II	Traj. PPA	0.57 [0.56-0.59]	0.59 [0.58-0.60]	0.59 [0.58-0.61]	0.63 [0.62-0.64]	0.65 [0.64-0.66]
	Traj. PPA + Sum. stats	0.60 [0.59-0.62]	0.66 [0.65-0.67]	0.68 [0.67-0.69]	0.70 [0.69-0.72]	0.71 [0.70-0.72]
	Traj. PPA + Sum. stats + BL	0.61 [0.59-0.62]	0.66 [0.65-0.67]	0.67 [0.66-0.68]	0.70 [0.69-0.72]	0.71 [0.69-0.72]
Exp. III	Traj. PPA	0.67 [0.62-0.72]	0.61 [0.56-0.66]	0.68 [0.64-0.72]	0.72 [0.68-0.75]	0.76 [0.73-0.79]
	Traj. PPA + Sum. stats	0.71 [0.67-0.76]	0.74 [0.70-0.78]	0.80 [0.77-0.84]	0.78 [0.74-0.82]	0.77 [0.72-0.81]
	Traj. PPA + Sum. stats + BL	0.74 [0.69-0.78]	0.74 [0.70-0.78]	0.81 [0.77-0.85]	0.79 [0.75-0.82]	0.76 [0.72-0.81]

Mean and 95% Confidence Interval limits are shown over 25 independent experimental repeats.

For Experiment II, predicting the presence of SCI, models showed lower performance in ROC-AUC than Experiment II in general, with model performance increasing over time and with the inclusion of summary statistics and baseline predictors (Table 3, Fig. 5b). For models using trajectory predictors alone, the performance in the out-of-train sample was low (day 1: 0.57 [0.56-0.59] to day 21: 0.65 [0.64-0.66]). The best models were those with trajectory and summary predictors (0.71 [0.70-0.72]), and trajectory, summary, and baseline predictors (0.71 [0.69-0.72]), including data up to 21 days. Interestingly, SAPS II alone did not perform better than a random guess, with an ROC-AUC for the out-of-train sample of 0.5 [0.49-0.52], and the addition of SAPS II to the baseline predictors did not provide any improvement in classification performance (Supplementary Fig. 11). In the SAPS II subset, the trajectory plus summary statistic predictor set reached an ROC-AUC for the out-of-train sample of 0.89 [0.88-0.90] (Supplementary Fig. 11).

Experiment III was set to classify whether SCI patients in the TRACK-SCI (not used during trajectory modeling) cohort presented motor complete (AIS A or B) vs. motor incomplete (AIS C, D or E) neurological deficits at the latest available in-hospital timepoint. With trajectory predictors alone, ROC-AUC improved from 0.67 [0.62-0.72] considering up to day 1 of lab data, to 0.76 [0.73–0.79] by day 21 (Table 3, Fig. 5c). The addition of lab marker summary statistics and baseline predictors to the trajectory PPAs increased performance for day 1 to 0.74 [0.69–0.78], with the best model achieved on day 7 (0.81 [0.77–0.85]).

A decline in performance was observed on the out-of-train sample across all time points compared to the performance on the training sample, potentially signaling overfitting. For Experiment I, the highest ROC-AUC dropped from 0.95 [0.94–0.95] in the training sample to 0.89 [0.88–0.89] in the out-of-train sample. In Experiment II, this drop went from 0.82 [0.82–0.82] to 0.71 [0.70–0.72], and for Experiment III, from 0.96 [0.94–0.97] to 0.81 [0.77–0.85]. Similar trends were observed for PR-AUC.

We also identified the most important predictors for each model using a predictor importance metric. Across the three experiments with different models, the blood biomarker PPA consistently emerged among the most important variables. This consistency highlights their relevance to predicting the SCI outcome of interest. For example, the PPAs of white blood cell count, potassium, chloride, MCV, and magnesium were among the important predictors of in-hospital mortality (Supplementary Figs. 14–16).

## Discussion

Secondary spinal cord damage triggers pathophysiological cascades of measurable events that are detectable in the blood^21,29^, as well as hematologic abnormalities that correlate with clinical metrics of injury severity^29,30^. Leister et al. ^22^. reported temporal changes in different blood metrics from a few hours, to weeks, to a year after SCI, predicting whether patients will walk one year after injury. Furthermore, combining blood markers in static prognostic models with clinical characteristics have been shown to improve prediction performance^14,18,19^. We demonstrated that longitudinal data changes from routinely collected in-hospital blood tests can be modeled, and their trajectories utilized as dynamic biomarkers in machine learning for diagnosis and prognosis in SCI. We also show that predictability improves as more lab data becomes available over time, showing the potential clinical value of a dynamic prediction tool.

By modeling multi-class trajectories of blood markers, we provide evidence that there are different temporal pattern trajectories after spine trauma and SCI and that those are, for the most part, non-linear and non-Gaussian responses. Our work distinguishes itself by tackling the multidimensionality, temporality, asynchrony, non-linearity, and heterogeneity inherent in routine blood data—a significant leap from previous studies that only touched on these issues in isolation, which may affect the utility of the models^31,32^. Considering these factors in the modeling requires a large sample size, which can be challenging in rare and near-rare diseases such as SCI. We demonstrated that model development using EHR is feasible and that these models can be generalized to external cohorts with utility for predicting tasks. This should open new exciting venues for SCI research and other medical contexts with low incidence, since the knowledge gained from EHR can be transferable.

The trajectories revealed distinct demographic and clinical characteristics. Chemical blood analytes—major electrolytes that regulate normal physiological function—can signal various pathophysiological events when dysregulated^33^. For each electrolyte, two distinct trajectories emerged: a low-membership class showed higher in-hospital mortality rates, characterized by non-linear changes with high dynamic range and above-normal values. They all associated with a higher average number of clinical diagnoses, serving as a potential surrogate measure for comorbidities and medical events. This pattern suggests distinct trajectories in severe patients who had a higher likelihood of hospital mortality compared to those with less physiological distress or better homeostatic regulation. This aligns with the common use of blood analytes in calculating patient severity scores^34^.

Regarding hematology values, hematocrit, hemoglobin, and red blood cells showed a high correlation with each other. The three trajectories found for each analyte displayed similar temporal trends. Trajectory class 1, showing high initial values and slow steady reduction, had the lowest proportion of patients with SCI and spine fracture, and the lowest mortality rate. Class 2 showed high initial levels followed by rapid decay by day 7, while class 3 maintained low values throughout the 21 days. Classes 2 and 3 for hematocrit, hemoglobin, and red blood cells showed higher mortality rates and higher proportions of SCI patients with spine fractures than class 1. The main demographic differences between classes 2 and 3 were that class 2 patients were younger and predominantly male. All hematocrit and red blood cell counts fell below normal population levels, consistent with previous findings in acute SCI^16^. White blood cell values generally exceeded normal levels, a previously documented phenomenon^16^, which is expected given the post-SCI inflammatory process and immune dysregulation^6,18^. Class 2, comprising the majority of patients, maintained levels at the high end of the normal range and showed lower mortality rates and fewer SCI fracture cases than classes 1 and 3. Class 1 patients were older and showed higher mortality rates and more diagnoses than class 3. Following SCI, different white blood cell populations show dynamic temporal changes in blood presence, responding to injury-triggered pathophysiological processes^18^. A global white blood cell count may therefore lack the resolution to describe these temporal patterns; examining individual cell type levels might better characterize this heterogeneity. Future research should address this limitation.

Using the posterior probability of the modeled class trajectories, we trained classification models with decent performance to detect in-hospital mortality as early as one day after hospital admission. Prediction performance increased as more temporal data was considered for predicting trajectories. This suggests that estimating the probability of determining which trajectory of blood marker changes patients will follow can be a predictor in dynamic prognostic neurological outcome models or patient mortality. We compared our prediction of in-hospital mortality to those provided by SAPS II, a widely used scoring system for assessing illness severity, which has been validated as a predictor of in-hospital mortality^35,36^ When compared to our models, SAPS II’s performance is most comparable to the model with trajectories, summary statistics, and baseline predictors, which incorporates an overlapping set of variables. As shown in the results, SAPS II exhibits high predictive power at day 1; however, as time progresses, our model outperforms it. The addition of SAPS II to our complete set of predictors produces the best-performing models, suggesting that there is non-overlapping information between SAPS II and our predictors.

Compared with other in-hospital mortality prediction studies, our models performed similarly. Nader et al. ^37^. developed the Spinal Cord Injury Risk Score (SCIRS) using age, early AIS, whether the injury was cervical, the Abbreviated Injury Scale^38^, and the AOSpine Injury classification system^39,40^ with a ROC-AUC of 0.85 on a validation cohort. Our models reached similar performance after incorporating 7 days of blood marker data alone, and outperformed after that. The authors compared SCIRS with the general trauma Injury Severity Score (ISS)^38^, which resulted in a ROC-AUC of only 0.7. Since SCIRS expands on the ISS methods by incorporating the Abbreviated Injury Scale, it suggests that ISS alone might not capture all the information necessary for predicting in-hospital mortality in SCI. The New Injury Severity Score^41,42^ has been shown to outperform the ISS in a general trauma population, which grants further investigation in SCI. In addition, a recent work by Khavandegar et al. ^43^, benchmarked nine different severity scores for predicting in-hospital outcomes for general trauma populations including the Trauma and Injury Severity Score (TRISS)^44^, and the New TRISS (NTRISS)^45^. Compared to ISS, which only uses features of trauma location and severity, TRISS and NTRISS add physiological predictors (e.g., Systolic blood pressure) to the scoring system. Given our results and the good performance of the SAPSII in predicting in-hospital mortality in SCI, and that physiological features hold predictive power for SCI outcomes^15^, further work could explore TRISS and NTRISS in SCI. Furthermore, other previously described predictors of mortality in SCI include age and the presence of comorbidities^46^. Future work could also be directed to a combination of baseline characteristics, such as those included in SCIRS, and physiological features like those in TRISS, with modeling dynamic markers.

Additionally, we fit models to predict whether a patient has an SCI after spine trauma, which could be useful for comatose or obtunded patients when voluntary motor or sensory function cannot be assessed or for rapid risk diagnosis in emergency or rural settings. Models predicting the presence of SCI had a moderate performance at best, achieved by day 14 to 21. Prediction performance was better in the case of predicting in-hospital mortality than the presence of SCI, likely because spine trauma is a significant traumatic event, usually associated with polytraumatic processes (e.g., non-spine fractures) that trigger multiple systemic pathophysiological changes. Central nervous system (CNS) proteins such as the Glial Fibrillary Acidic Protein (GFAP) have been shown to increase in blood early after neurological trauma^47–49^, including SCI, and can be used to predict severity. This includes the first FDA-authorized point-of-care blood test in mild TBI, based on determining GFAP and UCH-L1 (Ubiquitin C-terminal hydrolase L1) levels^50^. Thus, more CNS-specific biomarkers might be necessary for determining the presence of spinal cord damage after trauma. Nonetheless, our results are promising inasmuch as our models show acceptable performance in the training sample (ROC-AUC: 0.82 when considering the complete set of predictors by 14 days). Future work could further explore the use of routine blood values in combination with other routine data as quick biomarkers for assessing the risk of an SCI after trauma. The addition of other early predictors, such as the cause of injury (e.g., fall), could improve classification performance for this task.

We demonstrated that early prediction of marker trajectory in an external cohort (TRACK-SCI patients) has prediction utility. This signifies that dynamic models based on real-world data can be generalized to data collected in other contexts, such as different hospitals. We also found that predicting neurological deficits using only laboratory data is possible as early as day 1 and improves as more data is included over time in determining patient trajectory. We have previously shown that early gene expression data from peripheral white blood cells within hours of SCI holds predictive power for discriminating individuals with complete SCI (AIS A vs. other AIS, ROC-AUC = 0.86), and AIS D vs. other AIS (ROC-AUC = 0.93) during hospitalization^21^. In that work, we assessed the ROC-AUC on the training sample, for which we obtained comparable ROC-AUCs (0.88 [0.86-0.89] for day 1 using the trajectories alone, Supplementary Table 15). Thus, our results add to the evidence that early blood signatures after injury can serve as biomarkers in clinical prediction models. Nonetheless, the observed drop in prediction performance from the in-train to the out-of-train indicates the challenges in generalizing these models. Considering a dynamic prediction process, the observed increase in performance is a promising approach to capture the temporal changes in those markers. Early prediction of injury severity is clinically relevant for decision-making, yet it is a challenging task through neurological assessment alone. We show the potential to predict whether an injury is motor complete or incomplete with routine blood data early after injury, and an increase in prediction performance as time progresses.

These results mark the first step toward developing a lab-based, dynamic prediction tool for SCI. Future studies will focus on refining key inputs and methods for developing and implementing clinical-decision support tools for dynamic prediction.

There are a few limitations to the present work. We limited the analysis to the first 21 days post-injury due to a rapid decrease in sample size beyond this period. However, some patients stayed in hospital longer. Since the length of hospital stay in SCI is related to patient severity^51^, other trajectories may emerge when more extended periods are considered. Additionally, our focus was on the most common routine blood markers, potentially excluding other predictors like differential white blood cell counts, which have shown distinct associations with SCI^18^. These markers were not included due to sample size constraints. Furthermore, since we focused on routine blood markers available in real-world data, we did not include prominent protein biomarkers in neurotrauma, such as GFAP^47–49,52^. However, our methodology is adaptable for future inclusion of these and other markers, possibly enhancing predictive accuracy. Furthermore, a deeper analysis of the clinical features of each trajectory subpopulation could improve understanding of patient phenotypes and guide the development of better prediction models by identifying early predictors of progressive neurological deficit due to SCI and additional stratifying factors.

It is important to note that we observed a considerable drop in prediction performance when evaluating models in the training data compared to the out-of-train data, especially for Experiments II and III. This could signal problems of overfitting to the training data and potential drift in the population between the training and the out-of-train data. Our classification models using ElasticNet provide some intrinsic mechanisms to reduce the number of predictors considered^53,54^. However, we did not explicitly select the regularization parameters that provide the best feature reduction. As future work explores the dynamic information that routine blood markers must provide, robust approaches to overfitting should be considered. As acute management of patients evolves, continuous model updating could be a solution to reduce prediction performance due to distributional drift.

We modeled the latent space of the blood trajectories in a separate process from the prediction task, which might result in non-ideal latent variables to maximize prediction performance. Recent methodologies like SeqRisk^55^ that use neural network advances in latent modeling, such as variational autoencoders with the power of transformers to encode longitudinal information, could improve prediction performance. The advantage of such a method would be the incorporation of other temporal routine information in patient history beyond the lab values, increasing longitudinal context to dynamic patient management.

Finally, we observed substantial differences between both MIMIC datasets in cohort demographics and hospital stay characteristics. Although both MIMIC databases come from the same hospital, they have been collected at two different epochs, and the patients in the database are subject to temporal variations in incidence. To our knowledge, there is no systematic reason for differences in case distribution between MIMIC versions for SCI or spine trauma. One potential explanation is that MIMIC-III uses version 9 of ICD codes, while MIMIC-IV uses versions 9 and 10. There are meaningful differences in how traumatic SCI is coded between the two versions, and although we used the version mapping provided by MIMIC, these differences may affect case definitions and detection. In addition, while MIMIC-III only includes patients admitted to the ICU, MIMIC-IV also includes patients admitted to the emergency department, which could explain differences in case distribution. There are substantial differences in the represented populations and in the data structure between MIMIC-III and MIMIC-IV^26^.

In conclusion, we demonstrated the utility of modeling heterogeneous temporal trends of blood markers collected in real-world scenarios to predict diverse diagnostic and prognostic tasks in spine trauma and SCI patients. Longitudinal finite mixture models are powerful tools to describe non-linear, multi-class trajectories of blood markers, potentially capturing latent pathophysiological events. Given that SCI and other neurological pathologies evolve, studying the non-linear dynamic changes of any biomarker level is more valuable than considering predictions with a static cut-off level. The dynamic prediction is promising. By modeling marker trajectories, we can predict a patient’s future pathophysiological changes as early as one day after hospital arrival. This allows for predictive models in diagnostic and prognostic decision-making. These results suggest that real-time prediction models using in-hospital data could support the planning and execution of SCI patient management. Future work should focus on implementing these models.

## Methods

The *Consolidated Reporting Guidelines for Prognostic and Diagnostic Machine Learning Modeling Studies: Development and Validation*^56^ was used for reporting, and the checklist can be found as a supplement.

### Study population

Two versions of the MIMIC dataset (MIMIC-III 1.4 and the MIMIC-IV 1.0) were accessed under a data use agreement through the PhysioNet project^57^. We identified patients aged 15 years and older with traumatic SCI or spine trauma (vertebral fracture), based on the International Classification of Diseases (ICD9/ICD10) diagnostic codes for emergency admissions^58^. These include all codes of the series ICD9: 951, 953, 806 and 805, and ICD10: S110, S111, S112, S113, S114, S115, S116, S118, S119, S140, S141, S142, S210, S240, S241, S310, S311, S340, S341, S342, S343. For each patient, we selected their first hospital admission that matched these criteria. We harmonized patient demographic data into one dataset and categorized patients into three groups based on their diagnoses: SCI with vertebral fracture (SCI Fracture), SCI without vertebral fracture (SCI no Fracture), and spine trauma without SCI (Spine Trauma).

For the classification of trajectories in new patients, we use data from patients enrolled in the Transforming Research and Clinical Knowledge in Spinal Cord Injury (TRACK-SCI) study, a longitudinal observational acute cohort study at the Zuckerberg San Francisco General Hospital and the University of California San Francisco. TRACK-SCI collects highly granular in-hospital and post-hospitalization data, including laboratory assays and neurological outcomes as early as the day of injury. Data was received de-identified. In TRACK-SCI, International Standards for Neurological Classification of Spinal Cord Injury (ISNCSCI) exams are performed during the initial admission for all patients, either as part of clinical care or separately for the TRACK-SCI study, and at regular intervals including admission (day 0 = 0–23 hours from injury), every 48 hours until postinjury day 7, and at hospital discharge. We used the latest exam available at hospital discharge AIS grade. The lab markers data during hospitalization of a total of 137 subjects enrolled in the TRACK-SCI study as of April 2022 were extracted from the patients’ records, as well as age and gender at time of study enrollment.

We used de-identified data from 137 patients enrolled in the prospective TRACK-SCI study obtained through a collaboration agreement. Data collection and extraction protocols for the TRACK-SCI study were approved by the Institutional Research Board (IRB) at the University of California, San Francisco, including informed consent from the participants. For MIMIC, as stated in their publications^25,26^, the Institutional Review Board at Beth Israel Deaconess Medical Center (BIDMC) granted a waiver of informed consent and approved the sharing of the research resource. The Research Ethics Board at the University of Waterloo also approved this secondary analysis of de-identified data.

### Modeling data

#### Laboratory values

We extracted laboratory values during hospital stays and calculated the time from admission to laboratory sample collection. The laboratory values with missing Logical Observation Identifier Names and Codes (LOINC) were excluded. Among 413 distinct laboratory markers (157 hematology, 161 chemistry, 25 blood gases, and 70 unknown; Supplementary Table 4), we focused on the 20 most common markers, measured in 90–98% of patients, as the modeling set. These markers were all from hematology and chemistry, measured in blood. This set was pre-processed for outliers and limited to data within 21 days of admission to address the drop in available patient data over time. Temporal spaghetti and marginal density plots were generated to assess the amount of available data for the laboratory markers, characterize their underlying distributions, identify potential outliers, and detect non-linear trends. A data filter was applied to detect and remove observations highly likely to be outliers. First, observations with a recorded value of 0 were excluded, as these are biologically implausible for any of the modeled markers. Next, we applied a modified version of John Tukey’s rule for outlier detection, using an interquartile range (IQR)-based criterion. Specifically, we set the lower and upper thresholds at the 20th and 80th percentiles, respectively, rather than Tukey’s conventional 25th and 75th percentiles, to allow for a more flexible definition of outliers. This adjustment was made to avoid excessive exclusion of extreme values in skewed distributions, where the standard Tukey method may be overly restrictive^59^.

#### Demographics and Hospital stay characteristics

Demographics included age, gender, ethnicity, and insurance type. Stay details included the length of stay (in days), number of ICD diagnostics, admission and discharge locations, and admission type. For MIMIC-III and IV, patient age was derived by subtracting the data a patient is registered to the emergency (EDREGTIME) from the birth date (DOB). The actual date of birth is not accessible; instead, a shifted version is provided. This date shift is consistent across all timestamps for each patient, allowing for the calculation of age by subtracting the shifted date of birth from the time of admission. Gender did not require any data cleaning. The length of stay was determined by the difference between admission time and discharge time (DISCHTIME). To align the two versions of the MIMIC databases, ethnicity, insurance, admission type, and locations were standardized by merging similar categories to reduce information loss. Age and gender were also harmonized with TRACK-SCI dataset for consistency.

#### Validation data

Laboratory markers were harmonized to align TRACK-SCI with MIMIC; dynamic ranges were compared to ensure consistent scaling, and TRACK-SCI lab values underwent the same preprocessing as MIMIC. In TRACK-SCI, International Standards for Neurological Classification of Spinal Cord Injury (ISNCSCI) exams are performed multiple times during hospitalization. We used the latest exam available at hospital discharge to calculate the American Spinal Injury Association Impairment Severity (AIS)^60^ grade, a 5-point scale (A to E) measuring neurological impairment after SCI, as the outcome.

### Multi-class trajectory modeling

Following recommendations^61^, we first applied Latent Class Growth Analysis (LCGA), for each modeling set marker, determining the optimal number of classes ranging from 1 to 5 and evaluating linear and polynomial-growth trajectories over time. These recommendations suggest using restricted models such as LCGA to initially explore the heterogeneity in the trajectories, estimate the number of classes, find the proper model specifications, and perform initial exploration on non-linearities^61^. We implemented three link functions: a linear link for continuous Gaussian markers, and a quadratic I-spline with three knots or Beta density function for other continuous markers. A total of 900 models were initially specified. First, LCGA models were developed in R using the lcmm package without random effects^62^. Model selection was based on the Bayes Information Criterion (BIC)^63^, Integrated Complete-data Likelihood (ICL)^61^, and the Average Posterior Probability of Assignment (APPA)^64^, prioritizing lower BIC and ICL values and an APPA above 0.7. Each class was required to represent at least 20 subjects. After selecting plausible models based on the aforementioned criteria, we refined the LCGA results using Growth Mixture Models (GMMs) with random effects. To relax the symmetrical constraints of polynomial transformations, natural splines instead of polynomials were used to consider a non-linear basis for the time trajectories. The number of degrees for the splines was chosen from the number of degrees selected during the LCGA exploratory analysis. A total of 20 models were then selected^53^. Multi-class trajectory modeling and model selection were performed on MIMIC data, with the selected models validated using the TRACK-SCI dataset.

Plots of the model criteria for these models for red blood cell count and potassium are provided in Fig. 5. An interpretation of the model fit selection would go as follows. For red blood cell count, the polynomial degree is the dominant determinant of better model fit by both BIC and ICL. In these cases, we conclude that non-linear trends are a better fit and that the type of link function is almost irrelevant. This informs that assuming a Gaussian response is acceptable for markers with this pattern of model fit. Regarding the number of classes, for red blood cell count, BIC reduces progressively with the increase of classes, while ICL presents a more prominent drop at two classes but with a more stable value afterward. APPA has a more linear reduction as the number of classes increases. Given that APPA reached acceptable levels in most cases, we used BIC and ICL as the main decision-making tools for this initial step, leaving APPA as a secondary factor. Therefore, we concluded that a linear link and a polynomial of order 3 are best for this variable, selecting between 2 to 4 trajectory classes for modeling red blood cell count. In a different example, we can look at Potassium, where both the polynomial order and the shape of the link function are important for model fit. In this case, we selected models with polynomials of order 3, link function of type beta or spline, and 2 or 3 trajectory classes for further exploration.

In summary, most markers show that at least 2, with a median of 3, trajectory classes are needed to model the data, indicative of heterogeneity in the selected cohort. In addition, most models presented a better fit with a polynomial transformation of degree 2 or 3, indicative that non-linear trends are present in the data. Finally, with the exception of bicarbonate, chloride, hematocrit, hemoglobin, MCHC, and red blood cell count, all other marker data were better fitted to models with non-linear link functions (beta or I-spline), which suggests that blood markers are better modeled by non-Gaussian conditional distributions.

After model exploration, we selected those models that showed a better fit based on BIC, ICL, and APPA (Supplementary Table 5). There was a clear best choice for some markers, while the selection was more subjective for others. In those cases, we selected different models for the next step. We then went through a similar process, using GMMs, but now with the inclusion of the random effects. In addition, instead of polynomial transformations for the time variable, to relax some of the polynomial geometrical constrictions, we used a natural spline to model non-linear trends, with degrees determined through the exact search as before. A total of 122 models were specified. The posterior probability of class assignment (PPA) for each marker and subject was calculated, and class membership was assigned using maximum a posteriori probability estimates. Those posterior probabilities were used as predictors in an Elastic Net model for classification. PPA for each marker was high as early as 1 day of hospital arrival, with an average of 0.93 (SD: 0.12) across markers, classes, and timepoints (Supplementary Figs. 5, 6).

### Machine learning classifiers and outcomes

We designed three predictive modeling experiments using ElasticNet^53,54^ models (“glmnet” model in the R caret package)^65^ with increasing expected prediction difficulty: to predict in-hospital mortality (Experiment I), SCI occurrence in spine trauma patients (Experiment II), and SCI severity based on the last in-hospital AIS grade in the TRACK-SCI cohort (Experiment III). To simulate the incorporation of new data and dynamic prediction, we set data cutoffs at 1, 3, 7, 14, and 21 days post-injury. Predictors included the posterior probability of assignment (PPA) to a class trajectory for each subject and each marker, summary statistics (mean, standard deviation, minimum, and maximum) of marker values within the considered timeframe, and demographic variables at admission: age, gender, ethnicity, and insurance type (referred to as baseline predictors). For Experiment III, only age and gender were considered as baseline predictors. Model training considered three different predictor sets: PPA alone, PPA + summary statistics, and PPA + summary statistics + baseline predictors. Each experiment type and cutoff combination was set as a binary classification and run 25 times with varying random seeds, using a fixed seed across cutoffs for comparability. We used an 80/20 training/testing split. For hyperparameter tuning, we performed five-fold cross-validation (CV) independently for each of the 25 runs. ElasticNet requires two parameters, the mixing parameter alpha, and the regularization parameter lambda. We performed a gridsearch for alpha = {0.00, 0.11, 0.22, 0.33, 0.44, 0.55, 0.66, 0.77, 0.88, 1.00} and lambda = {0.0001, 0.11, 0.22, 0.33, 0.44, 0.55, 0.66, 0.77, 0.88, 1.00}. The best combination of parameters was selected as the models with the largest classification kappa metric in CV. Model performance for the best-tuned model was evaluated both in the training data (in-train) and testing data (out-of-train) using the area under the receiver operating characteristic area under the curve (ROC-AUC) as a primary metric and precision-recall curve (PR-AUC) as a secondary metric, calculated with the pROC R package^29^. We chose AUC from ROC curves as a primary metric to allow for comparisons across experiments and timepoints, and because of its prevalent use in the literature. We provided further PR-AUC (supplementary material) as it provides context to the prediction performance relative to the prevalence of the positive class. Differences in the interpretation of both metrics can be read elsewhere^66^. ElasticNet is a regularized regression method that can perform partial feature selection by shrinking coefficients, making it effective for handling highly correlated data. We applied it to assess the importance of variables across different predictor sets for each experiment.

We benchmarked model performance in Experiments I and II against the regressions on Simplified Acute Physiology Score (SAPS) II^35^. This score measures disease severity and is widely used in ICUs. It was originally developed in 1984, combines 13 physiological measures and the patient’s age to assess the mortality risk for ICU patients. By 1993, the model was updated to SAPS II, incorporating 17 factors in total: 12 physiological metrics, age, admission type (whether it was a scheduled surgery, an emergency surgery, or a medical reason), and three variables for pre-existing conditions (AIDS, metastatic cancer, and blood cancers). For the physiological variables, the worst value during the first 24 h of ICU admission is used for the calculation.3 SAPS II was further modified in 2005, and six more criteria for assessment upon admission, including age, gender, the duration of hospital stay before ICU admission, the patient’s location prior to ICU, the clinical reason for admission, and whether there was a drug overdose involved was added. The latest version is used in this article. For MIMIC-III, we compute scores for the selected cohort using SQL code publicly available on GitHub (https://github.com/MIT-LCP/mimic-code/blob/main/mimic-iii/concepts/severityscores/sapsii.sql). For MIMIC-IV, we used the equivalent script (https://github.com/MIT-LCP/mimic-code/blob/main/mimic-iv/concepts/score/sapsii.sql).

When comparing model performance, note that SAPS II incorporates baseline data, physiological metrics, and their summary statistics. Therefore, its performance should be evaluated against models that also integrate trajectories, summary statistics, and baseline information.

### Variable importance

We assessed variable importance based on the magnitude of these standardized coefficients, normalizing the scores so that the most significant variable receives a score of 100 relative to others. We show variables with a mean importance of more than 40 for each Experiment and cut-off in Supplementary Figs. 14–16.

### Statistics

Differences between trajectory groups and patient characteristics (age, gender, ethnicity, cohort group, length of hospital stay, whether the patient died in hospital, and the number of ICD diagnostics), were analyzed using ANOVA or t-test for continuous variables and Fisher exact test for categorical variables. *P*-values were adjusted for false discovery rate by the Benjamini-Hochberg method, and q-values (adjusted p values) were reported. The level of significance was set at q < 0.05. AUC values were represented with the average and 95% confidence intervals for the 25 independent repeats for each experimental set up.

## Supplementary information

Supplementary Material

## Data Availability

The MIMIC-III and MIMIC-IV datasets were accessed under a data use agreement through the PhysioNet project. Access to these datasets requires approval and completion of the required data use agreement. Anyone attempting to reproduce our work will need to gain access to these data. The TRACK-SCI dataset was obtained as part of a research collaboration and consists of de-identified data. Access to the final TRACK-SCI data that reproduces this work can be found in the Open Data Commons for Spinal Cord Injury (odc-sci.org) with DOI: 10.34945/F5PK6X.

## Code availability

The code used in this study was implemented in R and is available at https://github.com/ATE-DRIVEN-lab/blood_trajectory_modeling_EHR_SCI, and archived at 10.5281/zenodo.15347252.

## References

[1] James, S. L. et al. Global, regional, and national burden of traumatic brain injury and spinal cord injury, 1990–2016: a systematic analysis for the Global Burden of Disease Study 2016. *Lancet Neurol.* **18**, 56–87 (2019). 30497965 10.1016/S1474-4422(18)30415-0 PMC6291456

[2] Merritt, C. H., Taylor, M. A., Yelton, C. J. & Ray, S. K. Economic impact of traumatic spinal cord injuries in the United States. *Neuroimmunol. Neuroinflammation* **6** (2019). 10.20517/2347-8659.2019.15 PMC8052100 33869674

[3] Safdarian, M. et al. Global, regional, and national burden of spinal cord injury, 1990–2019: a systematic analysis for the Global Burden of Disease Study 2019. *Lancet Neurol.* **22**, 1026–1047 (2023). 37863591 10.1016/S1474-4422(23)00287-9 PMC10584692

[4] Failli, V. et al. Functional neurological recovery after spinal cord injury is impaired in patients with infections. *Brain: A J. Neurol.* **135**, 3238–3250 (2012). 10.1093/brain/aws267 23100450

[5] Fouad, K., Popovich, P. G., Kopp, M. A. & Schwab, J. M. The neuroanatomical-functional paradox in spinal cord injury. *Nat. Rev. Neurol.* **17**, 53–62 (2021). 33311711 10.1038/s41582-020-00436-x PMC9012488

[6] Jogia, T., Kopp, M. A., Schwab, J. M. & Ruitenberg, M. J. Peripheral white blood cell responses as emerging biomarkers for patient stratification and prognosis in acute spinal cord injury. *Curr. Opin. Neurol.* **34**, 796–803 (2021). 34608075 10.1097/WCO.0000000000000995 PMC8631147

[7] Liebscher, T. et al. Cervical spine injuries with acute traumatic spinal cord injury: spinal surgery adverse events and their association with neurological and functional outcome. *Spine* **47**, E16–E26 (2022). 34027924 10.1097/BRS.0000000000004124 PMC8654254

[8] Khorasanizadeh, M. et al. Neurological recovery following traumatic spinal cord injury: a systematic review and meta-analysis. *J. Neurosurg. Spine* 1–17. 10.3171/2018.10.SPINE18802 (2019). 10.3171/2018.10.SPINE18802 30771786

[9] Albayar, A. A. et al. Biomarkers in spinal cord injury: prognostic insights and future potentials. *Front. Neurol.* **10**, 27 (2019). 30761068 10.3389/fneur.2019.00027 PMC6361789

[10] Ruiz, I. A. et al. Incidence and natural progression of neurogenic shock after traumatic spinal cord injury. *J. Neurotrauma* **35**, 461–466 (2018). 29141498 10.1089/neu.2016.4947

[11] Haefeli, J. et al. Multivariate analysis of MRI biomarkers for predicting neurologic impairment in cervical spinal cord injury. *Am. J. Neuroradiol.* **38**, 648–655 (2017). 28007771 10.3174/ajnr.A5021 PMC5671488

[12] Talbott, J. F. et al. The Brain and Spinal Injury Center score: a novel, simple, and reproducible method for assessing the severity of acute cervical spinal cord injury with axial T2-weighted MRI findings. *J. Neurosurg. Spine* **23**, 495–504 (2015). 26161519 10.3171/2015.1.SPINE141033

[13] Hawryluk, G. et al. Mean arterial blood pressure correlates with neurological recovery after human spinal cord injury: analysis of high frequency physiologic data. *J. Neurotrauma* **32**, 1958–1967 (2015). 25669633 10.1089/neu.2014.3778 PMC4677564

[14] Squair, J. W. et al. Spinal cord perfusion pressure predicts neurologic recovery in acute spinal cord injury. *Neurology* **89**, 1660–1667 (2017). 28916535 10.1212/WNL.0000000000004519

[15] Torres-Espín, A. et al. Topological network analysis of patient similarity for precision management of acute blood pressure in spinal cord injury. *Elife* **10**, e68015 (2021). 34783309 10.7554/eLife.68015 PMC8639149

[16] Brown, S. J. et al. A PReliminary Cohort Study Assessing Routine Blood Analyte Levels and Neurological Outcome after Spinal Cord Injury. *J. Neurotrauma* **37**, 466–480 (2020). 31310157 10.1089/neu.2019.6495 PMC6978787

[17] Harrington, G. M. B. et al. Routinely measured hematological markers can help to predict american spinal injury association impairment scale scores after spinal cord injury. *J. Neurotrauma* **38**, 301–308 (2021). 32703074 10.1089/neu.2020.7144 PMC7826437

[18] Jogia, T. et al. Prognostic value of early leukocyte fluctuations for recovery from traumatic spinal cord injury. *Clin. Transl. Med.* **11**, e272 (2021). 33463065 10.1002/ctm2.272 PMC7805435

[19] Kwon, B. K. et al. Cerebrospinal fluid inflammatory cytokines and biomarkers of injury severity in acute human spinal cord injury. *J. Neurotrauma* **27**, 669–682 (2010). 20038240 10.1089/neu.2009.1080

[20] Kwon, B. K. et al. Neurochemical biomarkers in spinal cord injury. *Spinal Cord.* **57**, 819–831 (2019). 31273298 10.1038/s41393-019-0319-8

[21] Kyritsis, N. et al. Diagnostic blood RNA profiles for human acute spinal cord injury. *J. Exp. Med.* **218**, e20201795 (2021). 33512429 10.1084/jem.20201795 PMC7852457

[22] Leister, I. et al. Routine blood chemistry predicts functional recovery after traumatic spinal cord injury: a post hoc analysis. *Neurorehab. Neural Repair* **35**, 321–333 (2021). 10.1177/1545968321992328 33615895

[23] Chen, S.-H. et al. Altered peripheral profile of blood cells in Alzheimer disease: A hospital-based case-control study. *Medicine* **96**, e6843 (2017). 28538375 10.1097/MD.0000000000006843 PMC5457855

[24] Dong, X., Nao, J., Shi, J. & Zheng, D. Predictive Value of Routine Peripheral Blood Biomarkers in Alzheimer’s Disease. *Front. Aging Neurosci.* **11** (2019). 10.3389/fnagi.2019.00332 PMC6906180 31866854

[25] Johnson, A. E. W. et al. MIMIC-III, a freely accessible critical care database. *Sci. Data* **3**, 1–9 (2016). 10.1038/sdata.2016.35 PMC4878278 27219127

[26] Johnson, A. E. W. et al. MIMIC-IV, a freely accessible electronic health record dataset. *Sci. Data* **10**, 1 (2023). 36596836 10.1038/s41597-022-01899-x PMC9810617

[27] Tsolinas, R. E. et al. Transforming Research and Clinical Knowledge in Spinal Cord Injury (TRACK-SCI): an overview of initial enrollment and demographics. *Neurosurg. Focus* **48**, E6 (2020). 32357323 10.3171/2020.2.FOCUS191030

[28] Pickles, A. & Croudace, T. Latent mixture models for multivariate and longitudinal outcomes. *Stat. Methods Med. Res.* **19**, 271–289 (2010). 19608600 10.1177/0962280209105016

[29] Bourguignon, L. et al. Natural progression of routine laboratory markers after spinal trauma: a longitudinal, multi-cohort study. *J. Neurotrauma* **38**, 2151–2161 (2021). 33882712 10.1089/neu.2021.0012 PMC8309438

[30] Furlan, J. C., Krassioukov, A. V. & Fehlings, M. G. Hematologic abnormalities within the first week after acute isolated traumatic cervical spinal cord injury: a case-control cohort study. *Spine* **31**, 2674–2683 (2006). 17077735 10.1097/01.brs.0000244569.91204.01

[31] Jolliffe, I. T. & Cadima, J. Principal component analysis: a review and recent developments. *Philos. Trans. R. Soc. A: Math., Phys. Eng. Sci.* **374**, 20150202 (2016). 10.1098/rsta.2015.0202 PMC4792409 26953178

[32] Huie, J. R. et al. Testing a multivariate proteomic panel for traumatic brain injury biomarker discovery: a TRACK-TBI pilot study. *J. Neurotrauma* **36**, 100–110 (2019). 30084741 10.1089/neu.2017.5449 PMC6306686

[33] Balcı, A. K. et al. General characteristics of patients with electrolyte imbalance admitted to emergency department. *World J. Emerg. Med.* **4**, 113–116 (2013). 25215103 10.5847/wjem.j.issn.1920-8642.2013.02.005 PMC4129840

[34] Bouch, D. C. & Thompson, J. P. Severity scoring systems in the critically ill. *Continuing Educ. Anaesth. Crit. Care Pain.* **8**, 181–185 (2008).

[35] Le Gall, J.-R., Lemeshow, S. & Saulnier, F. A New Simplified Acute Physiology Score (SAPS II) Based on a European/North American Multicenter Study. *JAMA* **270**, 2957–2963 (1993). 8254858 10.1001/jama.270.24.2957

[36] Beck, D. H., Smith, G. B., Pappachan, J. V. & Millar, B. External validation of the SAPS II, APACHE II and APACHE III prognostic models in South England: a multicentre study. *Intensive Care Med.* **29**, 249–256 (2003). 12536271 10.1007/s00134-002-1607-9

[37] Fallah, N. et al. Development of a machine learning algorithm for predicting in-hospital and 1-year mortality after traumatic spinal cord injury. *Spine J.* **22**, 329–336 (2022). 34419627 10.1016/j.spinee.2021.08.003

[38] Greenspan, L., McLellan, B. A. & Greig, H. Abbreviated Injury Scale and Injury Severity Score: a scoring chart. *J. Trauma* **25**, 60–64 (1985). 3965737 10.1097/00005373-198501000-00010

[39] Vaccaro, A. R. et al. AOSpine subaxial cervical spine injury classification system. *Eur. Spine J.* **25**, 2173–2184 (2016). 25716661 10.1007/s00586-015-3831-3

[40] Vaccaro, A. R. et al. AOSpine thoracolumbar spine injury classification system: fracture description, neurological status, and key modifiers. *Spine (Philos. Pa 1976)* **38**, 2028–2037 (2013). 10.1097/BRS.0b013e3182a8a381 23970107

[41] Sutherland, A. G., Johnston, A. T. & Hutchison, J. D. The new injury severity score: better prediction of functional recovery after musculoskeletal injury. *Value Health* **9**, 24–27 (2006). 16441521 10.1111/j.1524-4733.2006.00077.x

[42] Lavoie, A., Moore, L., LeSage, N., Liberman, M. & Sampalis, J. S. The New Injury Severity Score: a more accurate predictor of in-hospital mortality than the Injury Severity Score. *J. Trauma* **56**, 1312–1320 (2004). 15211142 10.1097/01.ta.0000075342.36072.ef

[43] Khavandegar, A. et al. Comparison of nine trauma scoring systems in prediction of inhospital outcomes of pediatric trauma patients: a multicenter study. *Sci. Rep.* **14**, 7646 (2024). 38561381 10.1038/s41598-024-58373-4 PMC10985103

[44] Singh, J., Gupta, G., Garg, R. & Gupta, A. Evaluation of trauma and prediction of outcome using TRISS method. *J. Emerg. Trauma Shock* **4**, 446–449 (2011). 22090735 10.4103/0974-2700.86626 PMC3214498

[45] Domingues, C. et al. New Trauma and Injury Severity Score (TRISS) adjustments for survival prediction. *World J. Emerg. Surg.* **13**, 12 (2018). 29541155 10.1186/s13017-018-0171-8 PMC5840784

[46] Blex, C. et al. Baseline predictors of in-hospital mortality after acute traumatic spinal cord injury: data from a level I trauma center. *Sci. Rep.* **12**, 11420 (2022). 35794189 10.1038/s41598-022-15469-z PMC9259676

[47] Abdelhak, A. et al. Blood GFAP as an emerging biomarker in brain and spinal cord disorders. *Nat. Rev. Neurol.* **18**, 158–172 (2022). 35115728 10.1038/s41582-021-00616-3

[48] Okonkwo, D. O. et al. GFAP-BDP as an acute diagnostic marker in traumatic brain injury: results from the prospective transforming research and clinical knowledge in traumatic brain injury study. *J. Neurotrauma* **30**, 1490 (2013). 23489259 10.1089/neu.2013.2883 PMC3751263

[49] Korley, F. K. et al. Prognostic value of day-of-injury plasma GFAP and UCH-L1 concentrations for predicting functional recovery after traumatic brain injury in patients from the US TRACK-TBI cohort: an observational cohort study. *Lancet Neurol.* **21**, 803–813 (2022). 35963263 10.1016/S1474-4422(22)00256-3 PMC9462598

[50] Commissioner, O. of the. FDA authorizes marketing of first blood test to aid in the evaluation of concussion in adults. *FDA* https://www.fda.gov/news-events/press-announcements/fda-authorizes-marketing-first-blood-test-aid-evaluation-concussion-adults (2020).

[51] Jang, H. J., Park, J. & Shin, H.-I. Length of hospital stay in patients with spinal cord injury. *Ann. Rehab. Med.* **35**, 798 (2011). 10.5535/arm.2011.35.6.798 PMC3309389 22506208

[52] Stukas, S. et al. Association of CSF and serum neurofilament light and glial fibrillary acidic protein, injury severity, and outcome in spinal cord injury. *Neurology* **100**, e1221 (2023). 36599698 10.1212/WNL.0000000000206744 PMC10033160

[53] Friedman, J., Hastie, T. & Tibshirani, R. Regularization paths for generalized linear models via coordinate descent. *J. Stat. Softw.* **33**, 1 (2010). 20808728 PMC2929880

[54] Zou, H. & Hastie, T. Regularization and variable selection via the elastic net. *J. R. Stat. Soc. Ser. B: Stat. Methodol.* **67**, 301–320 (2005).

[55] Öğretir, M., Koskinen, M., Sinisalo, J., Renkonen, R. & Lähdesmäki, H. SeqRisk: Transformer-augmented latent variable model for improved survival prediction with longitudinal data. Preprint at 10.48550/arXiv.2409.12709 (2024).

[56] Klement, W. & El Emam, K. Consolidated reporting guidelines for prognostic and diagnostic machine learning modeling studies: development and validation. *J. Med Internet Res* **25**, e48763 (2023). 37651179 10.2196/48763 PMC10502599

[57] Goldberger, A. L. et al. PhysioBank, PhysioToolkit, and PhysioNet: components of a new research resource for complex physiologic signals. *circulation* **101**, e215–e220 (2000). 10851218 10.1161/01.cir.101.23.e215

[58] World Health Organization. *International Statistical Classification of Diseases and Related Health Problems: Alphabetical Index* Vol. 3 (World Health Organization, 2004).

[59] Seo, S. *A Review and Comparison of Methods for Detecting Outliers in Univariate Data Sets. Doctoral dissertation, University of Pittsburgh,* https://d-scholarship.pitt.edu/7948/ (2006).

[60] Roberts, T. T., Leonard, G. R. & Cepela, D. J. Classifications in brief: American Spinal Injury Association (ASIA) Impairment Scale. *Clin. Orthop. Relat. Res* **475**, 1499–1504 (2017). 27815685 10.1007/s11999-016-5133-4 PMC5384910

[61] van der Nest, G., Lima Passos, V., Candel, M. J. J. M. & van Breukelen, G. J. P. An overview of mixture modelling for latent evolutions in longitudinal data: modelling approaches, fit statistics and software. *Adv. Life Course Res.* **43**, 100323 (2020). 36726256 10.1016/j.alcr.2019.100323

[62] Proust-Lima, C., Philipps, V. & Liquet, B. Estimation of extended mixed models using latent classes and latent processes: the R Package lcmm. *J. Stat. Softw.* **78**, 1–56 (2017).

[63] Schwarz, G. Estimating the dimension of a model. *Ann. Stat.* **6**, 461–464 (1978).

[64] Nagin, D. *Group-Based Modeling of Development* (Harvard University Press, 2005).

[65] Kuhn, M. Caret: classification and regression training. *Astrophysics Source Code Library* ascl-1505 (2015).

[66] Davis, J. & Goadrich, M. The relationship between Precision-Recall and ROC curves. In: *Proc. 23rd international conference on Machine learning* 233–240 (Association for Computing Machinery, New York, NY, USA, 2006). 10.1145/1143844.1143874.

