# Supplementary material for: Modeling trajectories of routine blood tests as dynamic biomarkers for outcome in spinal cord injury
Source: NPJ Digit Med. 2025 Jul 22;8:470. doi: 10.1038/s41746-025-01782-0 (PMC12284016; doi:10.1038/s41746-025-01782-0)
Supplement: Supplementary file 1 — Supplementary Material [file 41746_2025_1782_MOESM1_ESM.pdf]

Supplementary Material

Supplementary Table 1. MIMIC-III vs. MIMIC-IC in demographic characteristics

Supplementary Table 2. MIMIC-III vs. MIMIC-IV in hospital stay characteristics

Supplementary Table 3. Summary characteristics for the TRACK-SCI cohort

Supplementary Table 4. Cross-tabulation of laboratory analytes per category and fluid sample

Supplementary Table 5. Summary of parameters for the selected GMM trajectory models

Supplementary Table 6. Univariate comparisons between clusters of Anion Gap and Chloride

Supplementary Table 7. Univariate comparisons between clusters of Glucose and Magnesium

Supplementary Table 8. Univariate comparisons between clusters of Phosphate and Potassium

Supplementary Table 9. Univariate comparisons between clusters of Sodium and Urea Nitrogen

Supplementary Table 10. Univariate comparisons between clusters of Hematocrit and Hemoglobin

Supplementary Table 11. Univariate comparisons between clusters of MCH and MCHC

Supplementary Table 12. Univariate comparisons between clusters of MCV and Platelet Count

Supplementary Table 13. Univariate comparisons between clusters of RDW and Red Blood Cells

Supplementary Table 14. Univariate comparisons between clusters of White blood cells

Supplementary Table 15. ROC AUC performance for the in train sample per experiment and feature set over the different time cutoffs

Supplementary Table 16. PR AUC performance for the in train sample per experiment and feature set over the different time cutoffs

Supplementary Table 17. PR AUC performance for the out-of-train sample per experiment and feature set over the different time cutoffs

Supplementary Table 18. PR AUC performance for the in train sample per experiment and feature set over the different time cutoffs for SAPS II

Supplementary Table 19. PR AUC performance for the out-of-train sample per experiment and feature set over the different time cutoffs for SAPS II

Supplementary Table 20. ROC AUC performance for the in train sample per experiment and feature set over the different time cutoffs for SAPSII

Supplementary Table 21. ROC AUC performance for the out-of-train sample per experiment and feature set over the different time cutoffs for SAPSII

Supplementary Figure 1. Length of stay distribution for MIMIC.

Supplementary Figure 2. Spaghetti plots for the raw data of the modeling set of analytes for MIMIC (20 most common).

Supplementary Figure 3. Spaghetti plots for the outlier-cleaned TRACK-SCI minimal set of laboratory analytes for the first 21 days after admission

Supplementary Figure 4. Average posterior probability of assignment (APPA) for each trajectory class and marker at each time point window in MIMIC

Supplementary Figure 5. Average posterior probability of assignment (APPA) for each trajectory class and marker at each time point window in TRACK SCI

Supplementary Figure 6. Example of model fit plots for two different types of model selection “patterns”

Supplementary Figure 7. ROC-AUC performance of dynamic predictions on the train data.

Supplementary Figure 8. PR-AUC performance of dynamic predictions on the train data

Supplementary Figure 9. PR-AUC performance of dynamic predictions on the out-of-train data

Supplementary Figure 10. ROC-AUC performance of dynamic predictions on the train data for ICU patients with SAPS II

Supplementary Figure 11. ROC-AUC performance of dynamic predictions on the out-of-train data for ICU patients with SAPS II

Supplementary Figure 12. PR-AUC performance of dynamic predictions on the train data for ICU patients with SAPS II

Supplementary Figure 13. PR-AUC performance of dynamic predictions on the out-train data for ICU patients with SAPS II

Supplementary Figure 14. Variable importance for models with posterior probability of trajectory classification only

Supplementary Figure 15. Variable importance for models with posterior probability of trajectory classification and summary statistics of biomarkers

Supplementary Figure 16. Variable importance for models with posterior probability of trajectory classification, summary statistics and baseline predictors

Supplementary Tables

Supplementary Table 1. MIMIC-III vs. MIMIC-IV in demographic characteristics

Characteristic	***MIMIC-III*** *N = 1,106[1]*	***MIMIC-IV*** *N = 1,509[1]*	***p-value[2]***	***q-value[3]***
Age	53 (35, 72)	70 (51, 84)	<0.001	**<0.001**
Male	740 (67%)	813 (54%)	<0.001	**<0.001**
Insurance			<0.001	**<0.001**
Medicaid	114 (10%)	90 (6.0%)		
Medicare	335 (30%)	696 (46%)		
Other	624 (56%)	723 (48%)		
Other Government	33 (3.0%)	0 (0%)		
Ethnicity			0.055	0.055
ASIAN	18 (1.8%)	31 (2.4%)		
BLACK/AFRICAN AMERICAN	47 (4.8%)	78 (6.1%)		
HISPANIC/LATINO	46 (4.7%)	54 (4.2%)		
MULTI RACE/ETHNICITY	6 (0.6%)	0 (0%)		
OTHER	47 (4.8%)	59 (4.6%)		
WHITE	817 (83%)	1,064 (83%)		
Unknown	125	223		
cohort_group			<0.001	**<0.001**
SCI_Fracture	231 (21%)	151 (10%)		
SCI_noFracture	49 (4.4%)	76 (5.0%)		
Spine Trauma	826 (75%)	1,282 (85%)		

[1] Median (Q1, Q3); n (%)

[2] Wilcoxon rank sum test; Fisher's Exact Test for Count Data; Fisher's Exact Test for Count Data with simulated p-value (based on 2000 replicates)

[3] False discovery rate correction for multiple testing

Supplementary Table 2. MIMIC-III vs. MIMIC-IV in hospital stay characteristics

Characteristic	***MIMIC-III*** *N = 1,106[1]*	***MIMIC-IV*** *N = 1,509[1]*	***p-value[2]***	***q-value[3]***
Length of stay (days)	9 (6, 16)	6 (4, 10)	<0.001	<0.001
Unknown	0	1		
Admission location			<0.001	<0.001
CLINIC REFERRAL	235 (21%)	93 (6.2%)		
EMERGENCY ROOM	863 (78%)	1,257 (84%)		

TRANSFER FROM HOSP	8 (0.7%)	119 (7.9%)		
TRANSFER FROM SNF	0 (0%)	3 (0.2%)		
WALK-IN/SELF REFERRAL	0 (0%)	26 (1.7%)		
Unknown	0	11		
Discharge location			<0.001	<0.001
ACUTE HOSPITAL	0 (0%)	13 (0.9%)		
AGAINST ADVICE	7 (0.6%)	6 (0.4%)		
DIED	108 (9.8%)	85 (5.9%)		
HOME	320 (29%)	447 (31%)		
HOSPICE	6 (0.5%)	16 (1.1%)		
ICF	1 (<0.1%)	0 (0%)		
LONG TERM CARE	48 (4.3%)	67 (4.6%)		
REHAB	466 (42%)	315 (22%)		
SHORT TERM CARE	11 (1.0%)	0 (0%)		
SKILLED NURSING FACILITY	114 (10%)	482 (33%)		
TRANSFER TO OTHER	25 (2.3%)	13 (0.9%)		
Unknown	0	65		
Number of ICD diagnostics	11 (8, 16)	16 (10, 22)	<0.001	<0.001
cohort_group			<0.001	<0.001
SCI_Fracture	231 (21%)	151 (10%)		
SCI_noFracture	49 (4.4%)	76 (5.0%)		
Spine Trauma	826 (75%)	1,282 (85%)		

[1] *Median (Q1, Q3); n (%)*

[2] *Wilcoxon rank sum test; Fisher's Exact Test for Count Data with simulated p-value (based on 2000 replicates)*

[3] *False discovery rate correction for multiple testing*

Supplementary Table 3. Summary characteristics for the TRACK-SCI cohort

**Characteristic**	**TRACK-SCI N = 137**[1]
Age	58 (40, 69)
Gender	
Female	40 (29%)
Male	97 (71%)
Latest in-hospital AIS	
A	25 (19%)
B	7 (5.4%)
C	20 (15%)
D	75 (58%)
E	3 (2.3%)
Unknown	7

[1]Median (Q1, Q3); n (%)

Supplementary Table 4. Cross-tabulation of laboratory analytes per category and fluid sample

**Fluid sample \ Category**	Blood Gas	Chemistry	Hematology	**Total**
Ascites	0	9	13	**22**
Blood	24	104	71	**199**
Cerebrospinal Fluid (CSF)	0	3	13	**16**
Joint Fluid	0	1	11	**12**
Other Body Fluid	1	9	14	**24**
Pleural	0	8	15	**23**
Urine	0	27	20	**47**
Unknown	0	0	0	**70**
**Total**	**25**	**161**	**157**	**413**

Supplementary Table 5. Summary of parameters for the selected GMM trajectory models

**Analyte**	**k**	**link**	**np**	**d**	**BIC**	**ICL**	**APPA**	**%class1**	**%class2**	**%class3**
Anion Gap	2	beta	22	p3	104781.70	99681.87	0.98	2.00	98.00	NA
Bicarbonate	1	linear	15	p3	111559.02	111559.02	1.00	100.00	NA	NA
Calcium, Total	1	linear	10	p2	24868.36	24868.36	1.00	100.00	NA	NA
Chloride	2	beta	22	p3	127905.22	122725.3	1.00	0.85	99.15	NA
Glucose	2	beta	11	p1	222867.98	218145.3	0.91	10.46	89.54	NA
Hematocrit	3	linear	25	p3	138572.13	134284.5	0.82	31.93	13.51	54.56
Hemoglobin	3	linear	25	p3	64672.17	60404.33	0.82	26.74	11.37	61.89
MCH	3	linear	18	p2	46805.77	41775.40	0.97	94.97	1.84	3.19
MCHC	2	linear	14	p2	59109.67	54145.65	0.95	96.54	3.46	NA
MCV	2	beta	16	p2	97687.94	92518.59	0.99	98.92	1.08	NA
Magnesium	2	3-quant-splines	23	p3	-4861.69	-9859.52	0.97	1.95	98.05	NA
Phosphate	2	beta	16	p2	50457.54	45400.07	0.99	2.74	97.26	NA
Platelet Count	3	beta	27	p3	248772.75	243815.0	0.95	1.96	2.50	95.54
Potassium	2	beta	22	p3	23972.63	18936.72	0.97	3.11	96.89	NA
RDW	3	beta	20	p2	34238.71	29400.81	0.93	8.99	87.09	3.92
Red Blood Cells	3	linear	25	p3	13818.27	9586.07	0.81	18.94	10.95	70.11
Sodium	2	beta	16	p2	129149.14	124014.2	0.99	97.35	2.65	NA
Urea Nitrogen	2	beta	16	p2	146168.70	141216.9	0.95	7.65	92.35	NA
White Blood Cells	3	beta	27	p3	109955.10	104834.1	0.98	0.85	98.08	1.08

Supplementary Table 6. Univariate comparisons between clusters of Anion Gap and Chloride

	**Anion Gap**				**Chloride**			
**Characteristic**	**1**<br>**N = 52**	**2**<br>**N = 2548**	**p-value**	**q-value**	**1**<br>**N = 22**	**2**<br>**N = 2578**	**p-value**	**q-value**
Age	78 (63, 86)	63 (43, 80)	<0.001	**<0.001**	28 (22, 57)	63 (44, 80)	<0.001	**<0.001**
Gender			0.5722	0.6447			0.2759	0.4633
F	19 (37%)	1,036 (41%)			6 (27%)	1,049 (41%)		
M	33 (63%)	1,512 (59%)			16 (73%)	1,529 (59%)		
Ethnicity			0.6447	0.6447			0.3513	0.4633
ASIAN	2 (4.7%)	47 (2.1%)			0 (0%)	49 (2.2%)		
BLACK	1 (2.3%)	124 (5.6%)			0 (0%)	125 (5.6%)		
HISPANIC	2 (4.7%)	96 (4.3%)			1 (6.7%)	97 (4.3%)		
MULTI RACE	0 (0%)	6 (0.3%)			0 (0%)	6 (0.3%)		
OTHER	1 (2.3%)	103 (4.7%)			2 (13%)	102 (4.6%)		
WHITE	37 (86%)	1,833 (83%)			12 (80%)	1,858 (83%)		
Unknown	9	339			7	341		
Cohort			0.041	0.0717			0.7466	0.7466
SCI Fracture	2 (3.8%)	380 (15%)			4 (18%)	378 (15%)		
SCI noFracture	3 (5.8%)	121 (4.7%)			1 (4.5%)	123 (4.8%)		
Spine Trauma	47 (90%)	2,047 (80%)			17 (77%)	2,077 (81%)		
Length of stay (days)	7 (5, 11)	7 (4, 13)	0.5536	0.6447	9 (4, 14)	7 (5, 12)	0.3971	0.4633
Unknown	0	1			0	1		
Died in hospital	20 (38%)	169 (6.6%)	<0.001	**<0.001**	14 (64%)	175 (6.8%)	<0.001	**<0.001**
Number of diagnostics	26 (17, 31)	13 (9, 19)	<0.001	**<0.001**	15 (9, 26)	13 (9, 20)	0.2887	0.4633

Supplementary Table 7. Univariate comparisons between clusters of Glucose and Magnesium

	Glucose				Magnesium			
**Characteristic**	**1<br>N = 272**	**2<br>N = 2328**	**p-value**	**q-value**	**1<br>N = 50**	**2<br>N = 2513**	**p-value**	**q-value**
Age	70 (56, 80)	62 (42, 80)	<0.001	**<0.001**	65 (49, 80)	63 (43, 80)	0.6655	0.9317
Gender			0.013	**0.0152**			1	1
F	91 (33%)	964 (41%)			20 (40%)	1,017 (40%)		
M	181 (67%)	1,364 (59%)			30 (60%)	1,496 (60%)		
Ethnicity			0.2694	0.2694			0.5002	0.8754
ASIAN	8 (3.5%)	41 (2.0%)			1 (2.7%)	48 (2.2%)		
BLACK	12 (5.2%)	113 (5.6%)			3 (8.1%)	121 (5.6%)		
HISPANIC	14 (6.1%)	84 (4.2%)			3 (8.1%)	94 (4.3%)		
MULTI RACE	0 (0%)	6 (0.3%)			0 (0%)	6 (0.3%)		
OTHER	14 (6.1%)	90 (4.4%)			2 (5.4%)	99 (4.5%)		
WHITE	181 (79%)	1,689 (83%)			28 (76%)	1,811 (83%)		
Unknown	43	305			13	334		
Cohort			0.001	**0.0014**			0.2329	0.5434
SCI Fracture	50 (18%)	332 (14%)			10 (20%)	371 (15%)		
SCI noFracture	23 (8.5%)	101 (4.3%)			4 (8.0%)	119 (4.7%)		
Spine Trauma	199 (73%)	1,895 (81%)			36 (72%)	2,023 (81%)		
Length of stay (days)	9 (5, 18)	7 (4, 12)	<0.001	**<0.001**	8 (5, 14)	7 (5, 13)	0.9031	1
Unknown	0	1			0	1		
Died in hospital	38 (14%)	151 (6.5%)	<0.001	**<0.001**	13 (26%)	176 (7.0%)	<0.001	**<0.001**
Number of diagnostics	17 (11, 26)	13 (9, 19)	<0.001	**<0.001**	20 (10, 27)	13 (9, 19)	0.0013	**0.0047**

Supplementary Table 8. Univariate comparisons between clusters of Phosphate and Potassium

	Phosphate				Potassium			
**Characteristic**	**1**<br>**N = 70**	**2**<br>**N = 2483**	**p-value**	**q-value**	**1**<br>**N = 81**	**2**<br>**N = 2521**	**p-value**	**q-value**
Age	71 (56, 82)	63 (43, 80)	0.0017	**0.004**	76 (61, 85)	63 (43, 80)	<0.001	**<0.001**
Gender			0.7118	0.9035			0.7308	0.7308
F	30 (43%)	1,003 (40%)			31 (38%)	1,024 (41%)		
M	40 (57%)	1,480 (60%)			50 (62%)	1,497 (59%)		
Ethnicity			0.9035	0.9035			0.3538	0.4954
ASIAN	2 (3.6%)	47 (2.2%)			2 (2.8%)	47 (2.2%)		
BLACK	2 (3.6%)	122 (5.7%)			1 (1.4%)	124 (5.7%)		
HISPANIC	2 (3.6%)	94 (4.4%)			1 (1.4%)	97 (4.4%)		
MULTI RACE	0 (0%)	6 (0.3%)			0 (0%)	6 (0.3%)		
OTHER	2 (3.6%)	98 (4.6%)			5 (7.0%)	99 (4.5%)		
WHITE	48 (86%)	1,783 (83%)			62 (87%)	1,810 (83%)		
Unknown	14	333			10	338		
Cohort			0.3083	0.5396			0.056	0.098
SCI Fracture	6 (8.6%)	372 (15%)			5 (6.2%)	377 (15%)		
SCI noFracture	4 (5.7%)	118 (4.8%)			3 (3.7%)	122 (4.8%)		
Spine Trauma	60 (86%)	1,993 (80%)			73 (90%)	2,022 (80%)		
Length of stay (days)	8 (4, 14)	7 (5, 13)	0.8049	0.9035	8 (5, 13)	7 (4, 12)	0.7017	0.7308
Unknown	0	1			0	1		
Died in hospital	25 (36%)	162 (6.5%)	<0.001	**<0.001**	15 (19%)	174 (6.9%)	<0.001	**0.0012**
Number of diagnostics	23 (16, 34)	13 (9, 19)	<0.001	**<0.001**	21 (14, 30)	13 (9, 19)	<0.001	**<0.001**

Supplementary Table 9. Univariate comparisons between clusters of Sodium and Urea Nitrogen

Characteristic	Sodium 1 N = 2531	Sodium 2 N = 69	Sodium p-value	Sodium q-value	Urea Nitrogen 1 N = 199	Urea Nitrogen 2 N = 2401	Urea Nitrogen p-value	Urea Nitrogen q-value
Age	63 (43, 80)	78 (66, 88)	<0.001	**<0.001**	80 (70, 88)	61 (42, 79)	<0.001	**<0.001**
Gender			<0.001	**<0.001**			1	1
F	1,008 (40%)	47 (68%)			81 (41%)	974 (41%)		
M	1,523 (60%)	22 (32%)			118 (59%)	1,427 (59%)		
Ethnicity			0.2949	0.344			0.063	0.0882
ASIAN	46 (2.1%)	3 (4.5%)			8 (4.4%)	41 (2.0%)		
BLACK	122 (5.6%)	3 (4.5%)			7 (3.9%)	118 (5.7%)		
HISPANIC	98 (4.5%)	0 (0%)			5 (2.8%)	93 (4.5%)		
MULTI RACE	6 (0.3%)	0 (0%)			0 (0%)	6 (0.3%)		
OTHER	102 (4.7%)	2 (3.0%)			3 (1.7%)	101 (4.9%)		
WHITE	1,812 (83%)	58 (88%)			157 (87%)	1,713 (83%)		
Unknown	345	3			19	329		
Cohort			0.0845	0.1182			0.081	0.0945
SCI Fracture	378 (15%)	4 (5.8%)			20 (10%)	362 (15%)		
SCI noFracture	121 (4.8%)	3 (4.3%)			13 (6.5%)	111 (4.6%)		
Spine Trauma	2,032 (80%)	62 (90%)			166 (83%)	1,928 (80%)		
Length of stay (days)	7 (5, 13)	6 (4, 11)	0.5637	0.5637	7 (4, 11)	7 (5, 13)	0.0187	**0.0326**
Unknown	1	0			0	1		
Died in hospital	179 (7.1%)	10 (14%)	0.0304	0.0532	36 (18%)	153 (6.4%)	<0.001	**<0.001**
Number of diagnostics	13 (9, 19)	17 (13, 27)	<0.001	**<0.001**	20 (13, 27)	13 (9, 19)	<0.001	**<0.001**

Supplementary Table 10. Univariate comparisons between clusters of Hematocrit and Hemoglobin

	Hematocrit					Hemoglobin				
**Characteristic**	**1**<br>**N = 834**	**2**<br>**N = 353**	**3**<br>**N = 1425**	**p value**	**q value**	**1**<br>**N = 696**	**2**<br>**N = 296**	**3**<br>**N = 1,611**	**p value**	**q value**
Age	65 (46, 83)	47 (29, 63)	67 (47, 81)	<0.001	**<0.001**	62 (43, 81)	45 (29, 60)	67 (48, 82)	<0.001	**<0.001**
Gender				<0.001	**<0.001**				<0.001	**<0.001**
F	295 (35%)	79 (22%)	686 (48%)			222 (32%)	59 (20%)	774 (48%)		
M	539 (65%)	274 (78%)	739 (52%)			474 (68%)	237 (80%)	837 (52%)		
Ethnicity				0.4813	0.4813				0.1804	0.1804
ASIAN	21 (2.8%)	5 (1.8%)	23 (1.9%)			17 (2.7%)	4 (1.7%)	28 (2.0%)		
BLACK	42 (5.5%)	12 (4.3%)	71 (5.8%)			32 (5.1%)	8 (3.4%)	85 (6.1%)		
HISPANIC	37 (4.9%)	17 (6.1%)	46 (3.7%)			31 (4.9%)	14 (6.0%)	53 (3.8%)		
MULTI RACE	1 (0.1%)	1 (0.4%)	4 (0.3%)			1 (0.2%)	1 (0.4%)	4 (0.3%)		
OTHER	31 (4.1%)	17 (6.1%)	58 (4.7%)			29 (4.6%)	18 (7.7%)	59 (4.2%)		
WHITE	625 (83%)	227 (81%)	1,026 (84%)			517 (82%)	189 (81%)	1,166 (84%)		
Unknown	77	74	197			69	62	216		
Cohort				<0.001	**<0.001**				<0.001	**<0.001**
SCI Fracture	93 (11%)	94 (27%)	195 (14%)			81 (12%)	70 (24%)	231 (14%)		
SCI noFracture	57 (6.8%)	5 (1.4%)	63 (4.4%)			50 (7.2%)	7 (2.4%)	68 (4.2%)		
Spine Trauma	684 (82%)	254 (72%)	1,167 (82%)			565 (81%)	219 (74%)	1,312 (81%)		
Length of stay (days)	5 (4, 9)	9 (6, 16)	8 (5, 14)	<0.001	**<0.001**	6 (4, 9)	8 (5, 16)	8 (5, 14)	<0.001	**<0.001**
Unknown	0	1	0			0	0	1	<0.001	**<0.001**
Died in hospital	39 (4.7%)	29 (8.2%)	121 (8.5%)	0.003	**0.0035**	24 (3.4%)	29 (9.8%)	136 (8.4%)	<0.001	**<0.001**
Number of diagnostics	12 (8, 18)	13 (9, 20)	14 (9, 20)	<0.001	**<0.001**	11 (8, 17)	13 (9, 20)	15 (9, 20)	<0.001	**<0.001**

Supplementary Table 11. Univariate comparisons between clusters of MCH and MCHC

	MCH					MCHC			
**Characteristic**	**1**<br>**N = 2472**	**2**<br>**N = 48**	**3**<br>**N = 83**	**p value**	**q value**	**1**<br>**N = 2513**	**2**<br>**N = 90**	**p value**	**q value**
Age	63 (43, 80)	74 (53, 83)	67 (47, 82)	<0.001	**0.0023**	62 (43, 80)	76 (62, 86)	<0.001	**<0.001**
Gender				0.2759	0.3218			0.0043	**0.0099**
F	1,002 (41%)	15 (31%)	38 (46%)			1,005 (40%)	50 (56%)		
M	1,470 (59%)	33 (69%)	45 (54%)			1,508 (60%)	40 (44%)		
Ethnicity				0.001	**0.0023**			0.3083	0.3083
ASIAN	44 (2.1%)	0 (0%)	5 (6.8%)			46 (2.1%)	3 (3.6%)		
BLACK	112 (5.2%)	1 (2.4%)	12 (16%)			116 (5.3%)	9 (11%)		
HISPANIC	89 (4.2%)	1 (2.4%)	8 (11%)			95 (4.4%)	3 (3.6%)		
MULTI RACE	6 (0.3%)	0 (0%)	0 (0%)			6 (0.3%)	0 (0%)		
OTHER	101 (4.7%)	1 (2.4%)	4 (5.4%)			102 (4.7%)	4 (4.8%)		
WHITE	1,789 (84%)	38 (93%)	45 (61%)			1,807 (83%)	65 (77%)		
Unknown	331	7	9			341	6		
Cohort				0.2134	0.2988			0.3013	0.3083
SCI Fracture	364 (15%)	9 (19%)	9 (11%)			373 (15%)	9 (10%)		
SCI noFracture	116 (4.7%)	1 (2.1%)	8 (9.6%)			119 (4.7%)	6 (6.7%)		
Spine Trauma	1,992 (81%)	38 (79%)	66 (80%)			2,021 (80%)	75 (83%)		
Length of stay (days)	7 (4, 13)	7 (5, 11)	7 (4, 12)	0.7488	0.7488	7 (4, 13)	7 (5, 12)	0.0863	0.1511
Unknown	1	0	0			1	0		
Died in hospital	180 (7.3%)	7 (15%)	2 (2.4%)	0.029	0.0507	180 (7.2%)	9 (10%)	0.2992	0.3083
Number of diagnostics	13 (9, 19)	16 (11, 23)	17 (12, 21)	<0.001	**0.0023**	13 (9, 19)	21 (17, 28)	<0.001	**<0.001**

Supplementary Table 12. Univariate comparisons between clusters of MCV and Platelet Count

	MCV				Platelet Count				
**Characteristic**	**1**<br>**N = 2575**	**2**<br>**N = 28**	**p value**	**q value**	**1**<br>**N = 51**	**2**<br>**N = 65**	**3**<br>**N = 2487**	**p value**	**q value**
Age	63 (43, 80)	75 (63, 84)	0.0037	**0.0085**	78 (60, 87)	50 (36, 67)	63 (43, 80)	<0.001	**<0.001**
Gender			0.8477	0.8477				<0.001	**0.0012**
F	1,043 (41%)	12 (43%)			36 (71%)	24 (37%)	995 (40%)		
M	1,532 (59%)	16 (57%)			15 (29%)	41 (63%)	1,492 (60%)		
Ethnicity			0.3683	0.5156				0.6102	0.6102
ASIAN	48 (2.1%)	1 (4.8%)			0 (0%)	1 (1.9%)	48 (2.2%)		
BLACK	125 (5.6%)	0 (0%)			3 (6.1%)	3 (5.8%)	119 (5.5%)		
HISPANIC	98 (4.4%)	0 (0%)			2 (4.1%)	3 (5.8%)	93 (4.3%)		
MULTI RACE	6 (0.3%)	0 (0%)			0 (0%)	0 (0%)	6 (0.3%)		
OTHER	104 (4.7%)	2 (9.5%)			0 (0%)	5 (9.6%)	101 (4.7%)		
WHITE	1,854 (83%)	18 (86%)			44 (90%)	40 (77%)	1,788 (83%)		
Unknown	340	7			2	13	332		
Cohort			0.6237	0.7276				0.0035	**0.0049**
SCI Fracture	377 (15%)	5 (18%)			7 (14%)	16 (25%)	359 (14%)		
SCI noFracture	125 (4.9%)	0 (0%)			7 (14%)	0 (0%)	118 (4.7%)		
Spine Trauma	2,073 (81%)	23 (82%)			37 (73%)	49 (75%)	2,010 (81%)		
Length of stay (days)	7 (4, 13)	7 (5, 9)	<0.001	**<0.001**	9 (4, 12)	20 (13, 27)	7 (4, 12)	<0.001	**<0.001**
Unknown	1	0			0	0	1		
Died in hospital	181 (7.0%)	8 (29%)	<0.001	**0.002**	4 (7.8%)	8 (12%)	177 (7.1%)	0.2514	0.2933
Number of diagnostics	13 (9, 19)	17 (10, 28)	0.051	0.0893	16 (13, 23)	17 (9, 24)	13 (9, 19)	<0.001	**0.0016**

Supplementary Table 13. Univariate comparisons between clusters of RDW and Red Blood Cells

	**RDW**					**Red Blood Cells**				
**Characteristic**	**1**<br>**N = 234**	**2**<br>**N = 2267**	**3**<br>**N = 102**	**p value**	**q value**	**1**<br>**N = 493**	**2**<br>**N = 285**	**3**<br>**N = 1825**	**p value**	**q value**
Age	74 (58, 85)	62 (42, 80)	57 (38, 80)	<0.001	**<0.001**	62 (41, 81)	43 (29, 59)	66 (48, 81)	<0.001	**<0.001**
Gender				0.0175	**0.0306**				<0.001	**<0.001**
F	115 (49%)	897 (40%)	43 (42%)			165 (33%)	61 (21%)	829 (45%)		
M	119 (51%)	1,370 (60%)	59 (58%)			328 (67%)	224 (79%)	996 (55%)		
Ethnicity				0.1209	0.1411				0.0415	**0.0415**
ASIAN	8 (3.7%)	39 (2.0%)	2 (2.7%)			10 (2.2%)	6 (2.7%)	33 (2.1%)		
BLACK	20 (9.3%)	103 (5.2%)	2 (2.7%)			31 (6.9%)	6 (2.7%)	88 (5.5%)		
HISPANIC	8 (3.7%)	87 (4.4%)	3 (4.0%)			26 (5.8%)	15 (6.7%)	57 (3.6%)		
MULTI RACE	0 (0%)	5 (0.3%)	1 (1.3%)			1 (0.2%)	1 (0.4%)	4 (0.3%)		
OTHER	6 (2.8%)	96 (4.9%)	4 (5.3%)			22 (4.9%)	16 (7.2%)	68 (4.3%)		
WHITE	172 (80%)	1,637 (83%)	63 (84%)			357 (80%)	179 (80%)	1,336 (84%)		
Unknown	20	300	27			46	62	239		
Cohort				0.084	0.1175				<0.001	**<0.001**
SCI Fracture	25 (11%)	334 (15%)	23 (23%)			45 (9.1%)	66 (23%)	271 (15%)		
SCI noFracture	10 (4.3%)	112 (4.9%)	3 (2.9%)			40 (8.1%)	9 (3.2%)	76 (4.2%)		
Spine Trauma	199 (85%)	1,821 (80%)	76 (75%)			408 (83%)	210 (74%)	1,478 (81%)		
Length of stay (days)	8 (5, 13)	7 (4, 12)	9 (6, 12)	0.3452	0.3452	5 (4, 9)	8 (5, 16)	8 (5, 13)	<0.001	**<0.001**
Unknown	0	1	0			0	0	1		
Died in hospital	31 (13%)	140 (6.2%)	18 (18%)	<0.001	**0.0012**	20 (4.1%)	25 (8.8%)	144 (7.9%)	0.0045	**0.0052**
Number of diagnostics	20 (14, 25)	13 (9, 19)	15 (10, 23)	<0.001	**<0.001**	12 (8, 17)	13 (9, 20)	14 (9, 20)	<0.001	**<0.001**

Supplementary Table 14. Univariate comparisons between clusters of White blood cells

Characteristic	White Blood Cells				
	**1**<br>**N = 22**	**2**<br>**N = 2553**	**3**<br>**N = 28**	**p value**	**q value***
Age	82 (72, 88)	63 (43, 80)	69 (57, 83)	0.0032	**0.0075**
Gender				0.7431	0.867
F	7 (32%)	1,037 (41%)	11 (39%)		
M	15 (68%)	1,516 (59%)	17 (61%)		
Ethnicity				0.9925	0.9925
ASIAN	0 (0%)	49 (2.2%)	0 (0%)		
BLACK	1 (5.9%)	123 (5.6%)	1 (4.0%)		
HISPANIC	0 (0%)	98 (4.4%)	0 (0%)		
MULTI RACE	0 (0%)	6 (0.3%)	0 (0%)		
OTHER	1 (5.9%)	104 (4.7%)	1 (4.0%)		
WHITE	15 (88%)	1,834 (83%)	23 (92%)		
Unknown	5	339	3		
Cohort				0.017	**0.0238**
SCI Fracture	5 (23%)	368 (14%)	9 (32%)		
SCI noFracture	0 (0%)	122 (4.8%)	3 (11%)		
Spine Trauma	17 (77%)	2,063 (81%)	16 (57%)		
Length of stay (days)	7 (3, 14)	7 (5, 13)	7 (4, 10)	0.0043	**0.0075**
Unknown	0	1	0		
Died in hospital	8 (36%)	172 (6.7%)	9 (32%)	<0.001	**0.0017**
Number of diagnostics	27 (16, 34)	13 (9, 19)	15 (11, 21)	<0.001	**0.0013**

Supplementary Table 15. ROC AUC performance for the in train sample per experiment and feature set over the different time cutoffs

Exp.	Feature set	ROC AUC <= 1 day	ROC AUC <= 3 days	ROC AUC <= 7 days	ROC AUC <= 14 days	ROC AUC <= 21 days
Exp. I	Traj. PPA	0.76 [0.76-0.76]	0.77 [0.77-0.78]	0.78 [0.78-0.79]	0.81 [0.80-0.81]	0.81 [0.81-0.81]
	Traj. PPA + Sum. stats	0.88 [0.88-0.88]	0.90 [0.90-0.91]	0.92 [0.92-0.93]	0.94 [0.93-0.94]	0.94 [0.94-0.94]
	Traj. PPA + Sum. stats + BL	0.91 [0.91-0.92]	0.94 [0.93-0.94]	0.95 [0.95-0.95]	0.95 [0.94-0.95]	0.95 [0.95-0.96]
Exp. II	Traj. PPA	0.61 [0.61-0.62]	0.64 [0.63-0.65]	0.63 [0.62-0.65]	0.66 [0.66-0.67]	0.69 [0.68-0.69]
	Traj. PPA + Sum. stats	0.72 [0.72-0.73]	0.77 [0.77-0.77]	0.79 [0.79-0.79]	0.80 [0.80-0.81]	0.81 [0.80-0.81]
	Traj. PPA + Sum. stats + BL	0.74 [0.74-0.75]	0.79 [0.78-0.79]	0.80 [0.80-0.80]	0.82 [0.82-0.82]	0.82 [0.82-0.82]
Exp. III	Traj. PPA	0.88 [0.86-0.89]	0.85 [0.83-0.86]	0.86 [0.84-0.87]	0.89 [0.88-0.90]	0.90 [0.89-0.92]
	Traj. PPA + Sum. stats	0.96 [0.94-0.98]	0.92 [0.91-0.94]	0.92 [0.91-0.93]	0.94 [0.92-0.95]	0.95 [0.93-0.96]
	Traj. PPA + Sum. stats + BL	0.96 [0.94-0.97]	0.94 [0.93-0.95]	0.92 [0.91-0.93]	0.94 [0.92-0.95]	0.94 [0.93-0.96]

Mean and 95% Confidence Interval limits are shown over 25 independent experimental repeats.

Supplementary Table 16. PR AUC performance for the in train sample per experiment and feature set over the different time cutoffs

Exp.	Feature set	PR AUC <= 1 day	Prev. <= 1 day	PR AUC <= 3 days	Prev. <= 3 days	PR AUC <= 7 days	Prev. <= 7 days	PR AUC <= 14 days	Prev. <= 14 days	PR AUC <= 21 days	Prev. <= 21 days
Exp. I	Traj. PPA	0.26 [0.26-0.27]	0.08	0.34 [0.33-0.35]	0.07	0.37 [0.36-0.38]	0.07	0.37 [0.36-0.37]	0.07	0.36 [0.35-0.37]	0.07
Exp. I	Traj. PPA + Sum. stats	0.55 [0.54-0.56]	0.08	0.61 [0.60-0.62]	0.07	0.63 [0.62-0.65]	0.07	0.70 [0.68-0.71]	0.07	0.70 [0.68-0.71]	0.07
Exp. I	Traj. PPA + Sum. stats + BL	0.61 [0.59-0.63]	0.06	0.66 [0.65-0.68]	0.06	0.69 [0.67-0.71]	0.06	0.70 [0.68-0.72]	0.06	0.74 [0.72-0.76]	0.06
Exp. II	Traj. PPA	0.22 [0.22-0.23]	0.16	0.23 [0.23-0.23]	0.15	0.25 [0.22-0.28]	0.15	0.27 [0.27-0.28]	0.15	0.29 [0.29-0.30]	0.15
Exp. II	Traj. PPA + Sum. stats	0.36 [0.35-0.38]	0.16	0.41 [0.41-0.42]	0.15	0.42 [0.42-0.43]	0.15	0.45 [0.44-0.45]	0.15	0.45 [0.44-0.45]	0.15
Exp. II	Traj. PPA + Sum. stats + BL	0.39 [0.37-0.40]	0.15	0.43 [0.43-0.44]	0.15	0.44 [0.43-0.44]	0.15	0.47 [0.46-0.48]	0.15	0.46 [0.46-0.47]	0.15
Exp. III	Traj. PPA	0.78 [0.75-0.81]	0.23	0.61 [0.58-0.64]	0.23	0.66 [0.63-0.68]	0.23	0.75 [0.73-0.77]	0.23	0.80 [0.78-0.82]	0.23
Exp. III	Traj. PPA + Sum. stats	0.94 [0.92-0.97]	0.23	0.84 [0.82-0.87]	0.23	0.83 [0.80-0.85]	0.23	0.81 [0.77-0.85]	0.23	0.86 [0.82-0.90]	0.23
Exp. III	Traj. PPA + Sum. stats + BL	0.94 [0.92-0.96]	0.23	0.88 [0.85-0.90]	0.23	0.83 [0.80-0.85]	0.23	0.81 [0.77-0.85]	0.23	0.85 [0.81-0.89]	0.23

Mean and 95% Confidence Interval limits are shown over 25 independent experimental repeats. Prev. = Prevalence of the positive class as a reference

Supplementary Table 17. PR AUC performance for the out-of-train sample per experiment and feature set over the different time cutoffs

Exp.	Feature set	PR AUC <= 1 day	Prev. <= 1 day	PR AUC <= 3 days	Prev. <= 3 days	PR AUC <= 7 days	Prev. <= 7 days	PR AUC <= 14 days	Prev. <= 14 days	PR AUC <= 21 days	Prev. <= 21 days
Exp. I	Traj. PPA	0.24 [0.22-0.25]	0.08	0.31 [0.28-0.33]	0.07	0.33 [0.30-0.35]	0.07	0.31 [0.29-0.34]	0.07	0.31 [0.28-0.33]	0.07
Exp. I	Traj. PPA + Sum. stats	0.30 [0.27-0.32]	0.08	0.44 [0.41-0.46]	0.07	0.48 [0.45-0.51]	0.07	0.52 [0.50-0.55]	0.07	0.52 [0.49-0.55]	0.07
Exp. I	Traj. PPA + Sum. stats + BL	0.31 [0.28-0.34]	0.06	0.36 [0.33-0.39]	0.06	0.40 [0.37-0.43]	0.06	0.46 [0.42-0.49]	0.06	0.47 [0.43-0.50]	0.06

Exp. II	Traj. PPA	0.20 [0.19-0.21]	0.16	0.20 [0.19-0.20]	0.15	0.22 [0.19-0.25]	0.15	0.23 [0.22-0.24]	0.15	0.26 [0.25-0.27]	0.15
Exp. II	Traj. PPA + Sum. stats	0.22 [0.21-0.24]	0.16	0.27 [0.26-0.28]	0.15	0.28 [0.27-0.29]	0.15	0.30 [0.28-0.31]	0.15	0.30 [0.29-0.32]	0.15
Exp. II	Traj. PPA + Sum. stats + BL	0.23 [0.22-0.24]	0.15	0.26 [0.25-0.28]	0.15	0.26 [0.24-0.27]	0.15	0.29 [0.27-0.30]	0.15	0.28 [0.27-0.30]	0.15
Exp. III	Traj. PPA	0.49 [0.43-0.56]	0.26	0.35 [0.29-0.40]	0.25	0.40 [0.35-0.45]	0.25	0.45 [0.40-0.50]	0.25	0.58 [0.53-0.63]	0.25
Exp. III	Traj. PPA + Sum. stats	0.53 [0.46-0.61]	0.26	0.51 [0.44-0.57]	0.25	0.58 [0.52-0.65]	0.25	0.51 [0.45-0.56]	0.25	0.51 [0.45-0.58]	0.25
Exp. III	Traj. PPA + Sum. stats + BL	0.56 [0.49-0.63]	0.26	0.50 [0.44-0.57]	0.25	0.58 [0.51-0.64]	0.25	0.53 [0.48-0.58]	0.25	0.53 [0.46-0.60]	0.25

Mean and 95% Confidence Interval limits are shown over 25 independent experimental repeats. Prev. = Prevalence of the positive class as a reference

Supplementary Table 18. PR AUC performance for the in train sample per experiment and feature set over the different time cutoffs for SAPS II

Exp.	Feature set	PR AUC <= 1 day	Prev. <= 1 day	PR AUC <= 3 days	Prev. <= 3 days	PR AUC <= 7 days	Prev. <= 7 days	PR AUC <= 14 days	Prev. <= 14 days	PR AUC <= 21 days	Prev. <= 21 days
Exp. I	SAPSII	0.44 [0.43-0.45]	0.11	0.44 [0.43-0.45]	0.11	0.44 [0.43-0.45]	0.11	0.44 [0.43-0.45]	0.11	0.44 [0.43-0.45]	0.11
Exp. I	Traj. PPA	0.32 [0.31-0.32]	0.11	0.41 [0.41-0.42]	0.11	0.44 [0.44-0.45]	0.11	0.43 [0.42-0.44]	0.11	0.42 [0.41-0.43]	0.11
Exp. I	Traj. PPA + Sum. stats	0.58 [0.56-0.59]	0.11	0.65 [0.63-0.66]	0.11	0.70 [0.69-0.72]	0.11	0.75 [0.74-0.77]	0.11	0.76 [0.74-0.77]	0.11
Exp. I	Traj. PPA + Sum. stats + BL	0.67 [0.65-0.69]	0.09	0.77 [0.75-0.79]	0.09	0.75 [0.73-0.77]	0.09	0.77 [0.76-0.79]	0.09	0.82 [0.80-0.84]	0.09
Exp. I	Traj. PPA + Sum. stats + BL + SAPSII	0.73 [0.71-0.74]	0.09	0.77 [0.75-0.79]	0.09	0.79 [0.77-0.80]	0.09	0.80 [0.79-0.81]	0.09	0.84 [0.82-0.85]	0.09
Exp. II	SAPSII	0.21 [0.21-0.21]	0.20	0.21 [0.21-0.21]	0.20	0.21 [0.21-0.21]	0.20	0.21 [0.21-0.21]	0.20	0.21 [0.21-0.21]	0.20
Exp. II	Traj. PPA	0.27 [0.26-0.27]	0.20	0.30 [0.27-0.33]	0.20	0.32 [0.29-0.36]	0.20	0.34 [0.33-0.34]	0.20	0.34 [0.33-0.35]	0.20
Exp. II	Traj. PPA + Sum. stats	0.61 [0.60-0.63]	0.11	0.67 [0.66-0.68]	0.11	0.72 [0.70-0.73]	0.11	0.77 [0.75-0.78]	0.11	0.79 [0.78-0.81]	0.11
Exp. II	Traj. PPA + Sum. stats + BL	0.43 [0.42-0.45]	0.20	0.51 [0.49-0.52]	0.20	0.51 [0.50-0.52]	0.20	0.55 [0.54-0.56]	0.20	0.53 [0.53-0.54]	0.20
Exp. II	Traj. PPA + Sum. stats + BL + SAPSII	0.45 [0.44-0.46]	0.20	0.51 [0.50-0.52]	0.20	0.51 [0.50-0.52]	0.20	0.55 [0.54-0.56]	0.20	0.53 [0.53-0.54]	0.20

Mean and 95% Confidence Interval limits are shown over 25 independent experimental repeats. Prev. = Prevalence of the positive class as a reference

Supplementary Table 19. PR AUC performance for the out-of-train sample per experiment and feature set over the different time cutoffs for SAPS II

Exp.	Feature set	PR AUC <= 1 day	Prev. <= 1 day	PR AUC <= 3 days	Prev. <= 3 days	PR AUC <= 7 days	Prev. <= 7 days	PR AUC <= 14 days	Prev. <= 14 days	PR AUC <= 21 days	Prev. <= 21 days
Exp. I	SAPSII	0.45 [0.42-0.49]	0.11	0.45 [0.42-0.49]	0.11	0.45 [0.42-0.49]	0.11	0.45 [0.42-0.49]	0.11	0.45 [0.42-0.49]	0.11
Exp. I	Traj. PPA	0.24 [0.22-0.26]	0.11	0.33 [0.31-0.35]	0.11	0.38 [0.36-0.41]	0.11	0.38 [0.36-0.41]	0.11	0.37 [0.35-0.39]	0.11

Exp. I	Traj. PPA + Sum. stats	0.34 [0.32-0.37]	0.11	0.45 [0.42-0.47]	0.11	0.48 [0.45-0.51]	0.11	0.54 [0.51-0.57]	0.11	0.56 [0.53-0.59]	0.11
Exp. I	Traj. PPA + Sum. stats + BL	0.34 [0.30-0.38]	0.09	0.43 [0.39-0.47]	0.09	0.49 [0.45-0.52]	0.09	0.49 [0.45-0.53]	0.09	0.51 [0.47-0.55]	0.09
Exp. I	Traj. PPA + Sum. stats + BL + SAPSII	0.41 [0.37-0.46]	0.09	0.48 [0.44-0.52]	0.09	0.54 [0.50-0.58]	0.09	0.57 [0.53-0.60]	0.09	0.57 [0.53-0.61]	0.09
Exp. II	SAPSII	0.21 [0.20-0.22]	0.20	0.21 [0.20-0.22]	0.20	0.21 [0.20-0.22]	0.20	0.21 [0.20-0.22]	0.20	0.21 [0.20-0.22]	0.20
Exp. II	Traj. PPA	0.23 [0.22-0.24]	0.20	0.24 [0.21-0.27]	0.20	0.28 [0.24-0.32]	0.20	0.29 [0.27-0.30]	0.20	0.29 [0.28-0.31]	0.20
Exp. II	Traj. PPA + Sum. stats	0.40 [0.38-0.42]	0.11	0.51 [0.48-0.54]	0.11	0.55 [0.52-0.57]	0.11	0.58 [0.56-0.61]	0.11	0.59 [0.57-0.62]	0.11
Exp. II	Traj. PPA + Sum. stats + BL	0.24 [0.23-0.26]	0.20	0.29 [0.27-0.30]	0.20	0.28 [0.27-0.29]	0.20	0.32 [0.31-0.34]	0.20	0.30 [0.29-0.31]	0.20
Exp. II	Traj. PPA + Sum. stats + BL + SAPSII	0.25 [0.24-0.27]	0.20	0.29 [0.27-0.30]	0.20	0.28 [0.27-0.29]	0.20	0.32 [0.31-0.33]	0.20	0.30 [0.29-0.31]	0.20

Mean and 95% Confidence Interval limits are shown over 25 independent experimental repeats. Prev. = Prevalence of the positive class as a reference

Supplementary Table 20. ROC AUC performance for the in train sample per experiment and feature set over the different time cutoffs for SAPSII

Exp.	Feature set	ROC AUC <= 1 day	ROC AUC <= 3 days	ROC AUC <= 7 days	ROC AUC <= 14 days	ROC AUC <= 21 days
Exp. I	SAPSII	0.85 [0.84-0.85]	0.85 [0.84-0.85]	0.85 [0.84-0.85]	0.85 [0.84-0.85]	0.85 [0.84-0.85]
	Traj. PPA	0.76 [0.76-0.76]	0.79 [0.78-0.79]	0.80 [0.80-0.81]	0.81 [0.81-0.82]	0.82 [0.81-0.82]
	Traj. PPA + Sum. stats	0.87 [0.87-0.88]	0.90 [0.90-0.90]	0.93 [0.92-0.93]	0.94 [0.93-0.94]	0.94 [0.93-0.94]
	Traj. PPA + Sum. stats + BL	0.92 [0.91-0.92]	0.95 [0.94-0.95]	0.96 [0.95-0.96]	0.95 [0.95-0.96]	0.96 [0.95-0.96]
	Traj. PPA + Sum. stats + BL + SAPSII	0.93 [0.93-0.93]	0.95 [0.95-0.96]	0.96 [0.96-0.97]	0.96 [0.95-0.96]	0.96 [0.96-0.97]
Exp. II	SAPSII	0.52 [0.51-0.52]	0.52 [0.51-0.52]	0.52 [0.51-0.52]	0.52 [0.51-0.52]	0.52 [0.51-0.52]
	Traj. PPA	0.61 [0.60-0.61]	0.63 [0.61-0.64]	0.62 [0.60-0.63]	0.65 [0.64-0.65]	0.67 [0.66-0.67]
	Traj. PPA + Sum. stats	0.88 [0.87-0.88]	0.90 [0.90-0.90]	0.93 [0.93-0.93]	0.94 [0.93-0.94]	0.94 [0.94-0.95]
	Traj. PPA + Sum. stats + BL	0.74 [0.72-0.75]	0.77 [0.77-0.78]	0.78 [0.78-0.79]	0.81 [0.80-0.81]	0.81 [0.80-0.81]
	Traj. PPA + Sum. stats + BL + SAPSII	0.75 [0.74-0.76]	0.78 [0.77-0.78]	0.79 [0.78-0.79]	0.81 [0.81-0.81]	0.81 [0.81-0.81]

Mean and 95% Confidence Interval limits are shown over 25 independent experimental repeats.

Supplementary Table 21. ROC AUC performance for the out-of-train sample per experiment and feature set over the different time cutoffs for SAPSII

Exp.	Feature set	ROC AUC <= 1 day	ROC AUC <= 3 days	ROC AUC <= 7 days	ROC AUC <= 14 days	ROC AUC <= 21 days
Exp. I	SAPSII	0.85 [0.84-0.86]	0.85 [0.84-0.86]	0.85 [0.84-0.86]	0.85 [0.84-0.86]	0.85 [0.84-0.86]
	Traj. PPA	0.69 [0.67-0.70]	0.72 [0.71-0.74]	0.75 [0.73-0.77]	0.78 [0.76-0.79]	0.78 [0.76-0.79]
	Traj. PPA + Sum. stats	0.78 [0.77-0.79]	0.82 [0.81-0.83]	0.84 [0.83-0.85]	0.87 [0.86-0.88]	0.88 [0.87-0.89]
	Traj. PPA + Sum. stats + BL	0.79 [0.77-0.81]	0.82 [0.80-0.84]	0.87 [0.85-0.88]	0.86 [0.85-0.88]	0.87 [0.85-0.88]
	Traj. PPA + Sum. stats + BL + SAPSII	0.84 [0.82-0.86]	0.85 [0.83-0.87]	0.88 [0.87-0.90]	0.89 [0.87-0.90]	0.89 [0.87-0.91]
Exp. II	SAPSII	0.50 [0.49-0.52]	0.50 [0.49-0.52]	0.50 [0.49-0.52]	0.50 [0.49-0.52]	0.50 [0.49-0.52]
	Traj. PPA	0.56 [0.54-0.57]	0.56 [0.54-0.57]	0.57 [0.56-0.58]	0.60 [0.58-0.61]	0.62 [0.61-0.64]
	Traj. PPA + Sum. stats	0.79 [0.77-0.80]	0.83 [0.82-0.84]	0.88 [0.87-0.88]	0.88 [0.87-0.89]	0.89 [0.88-0.90]
	Traj. PPA + Sum. stats + BL	0.56 [0.54-0.57]	0.61 [0.59-0.62]	0.62 [0.60-0.63]	0.65 [0.64-0.66]	0.65 [0.64-0.66]
	Traj. PPA + Sum. stats + BL + SAPSII	0.57 [0.56-0.58]	0.61 [0.59-0.62]	0.61 [0.60-0.63]	0.65 [0.64-0.66]	0.65 [0.64-0.66]

Mean and 95% Confidence Interval limits are shown over 25 independent experimental repeats.

Supplementary Figures

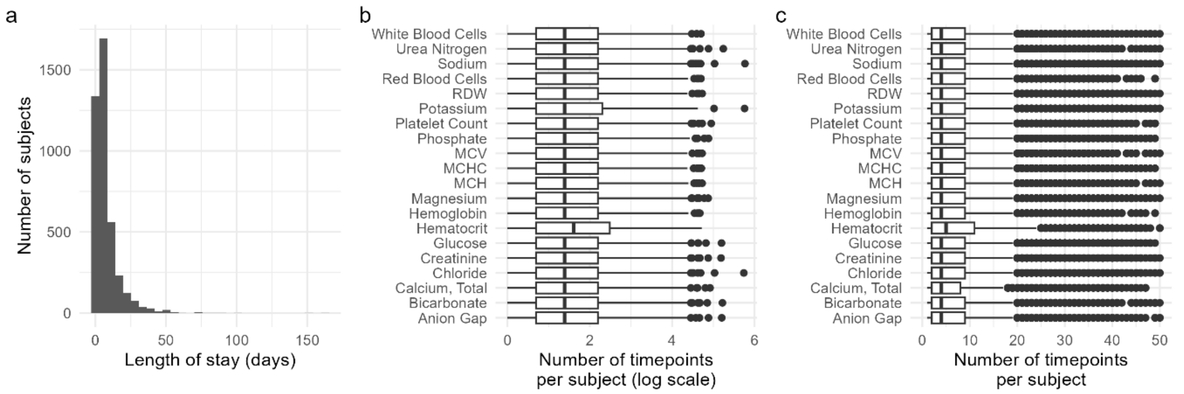

Supplementary Figure 1. Length of stay distribution for MIMIC. (a) Histogram of the length of stay in days for the cohort. (b) Boxplot of the logarithmic scale count of the number of measurements per subject and the modeling set of analytes (black dots are distributional outliers given the 1.5 X interquartile range commonly performed method). (c) Boxplot of the count of the number of measurements per subject and the modeling set of analytes (black dots are distributional outliers given the 1.5 X interquartile range commonly performed method). The number of subjects with data in a given marker also reduces over time, with very few subjects with data beyond 50 days (Supplementary Figure 1). This can also be confirmed by the distribution of length of stay, where the median time is 4.6 days (first quartile: 2.09 and third quartile: 9.07), and 96.4% of the cohort was discharged before 18 days in the hospital. These observations were used to justify the modeling time window of the first 3 weeks from hospitalization.

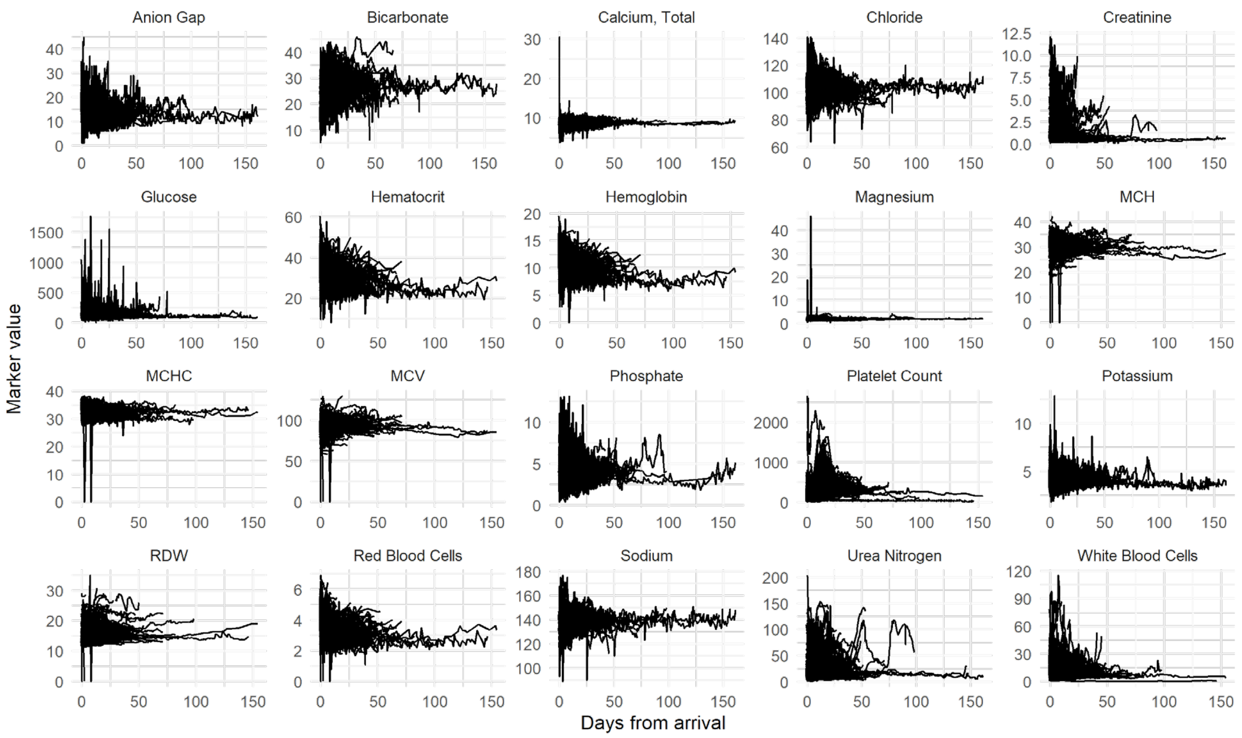

Supplementary Figure 2. Spaghetti plots for the raw data of the modeling set of analytes for MIMIC (20 most common). Note that unexpected spikes are observed, probably indicative of data errors. Each line represents a subject. As expected by the nature of the data, time is asynchronous, meaning that markers were obtained for each patient at different timepoints in non-regular intervals. No apparent trends are observable from the plots. We can observe fluctuations over time, with, in general, a high dynamic range early after admission that reduces as time progresses.

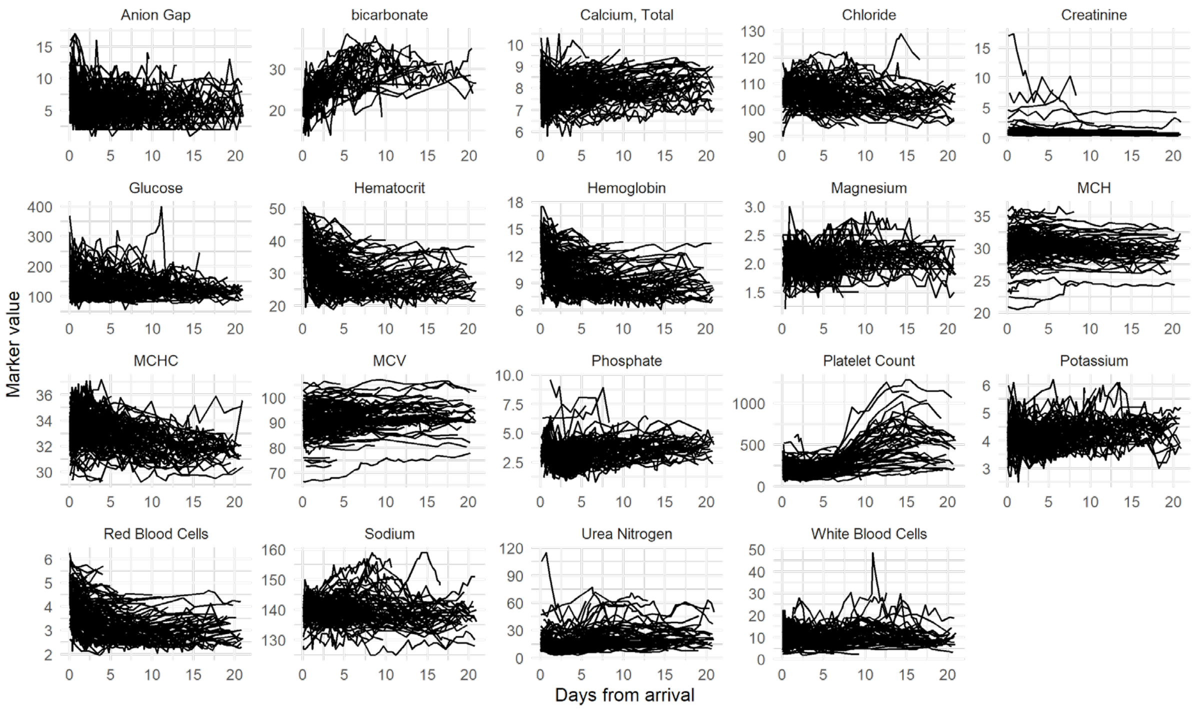

Supplementary Figure 3. Spaghetti plots for the outlier-cleaned TRACK-SCI minimal set of laboratory analytes for the first 21 days after admission. Each line represents a single subject.

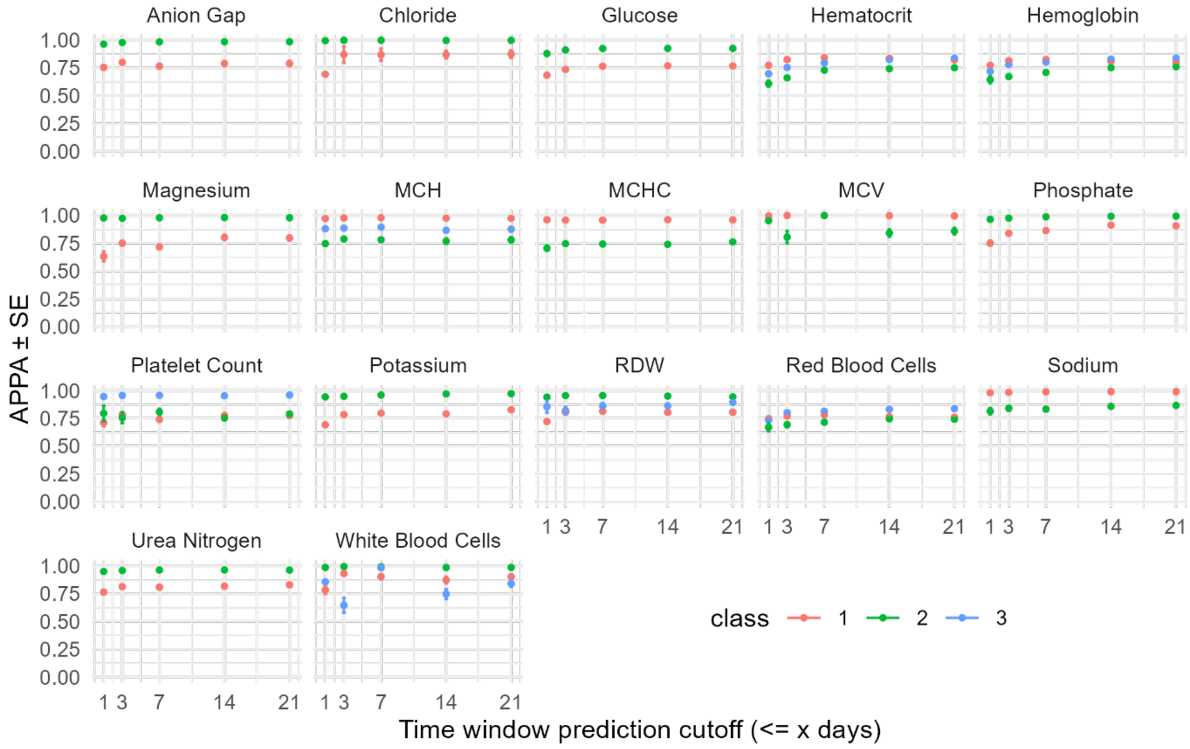

Supplementary Figure 4. Average posterior probability of assignment (APPA) for each trajectory class and marker at each time point window in MIMIC. Each point represents the APPA of using the respective model and <= x days of data to predict trajectory membership, where x is the time window cutoff. The standard error of the mean (SE) across PPA for all subjects is represented. Markers with a single trajectory class are not shown as they have an APPA = 1.

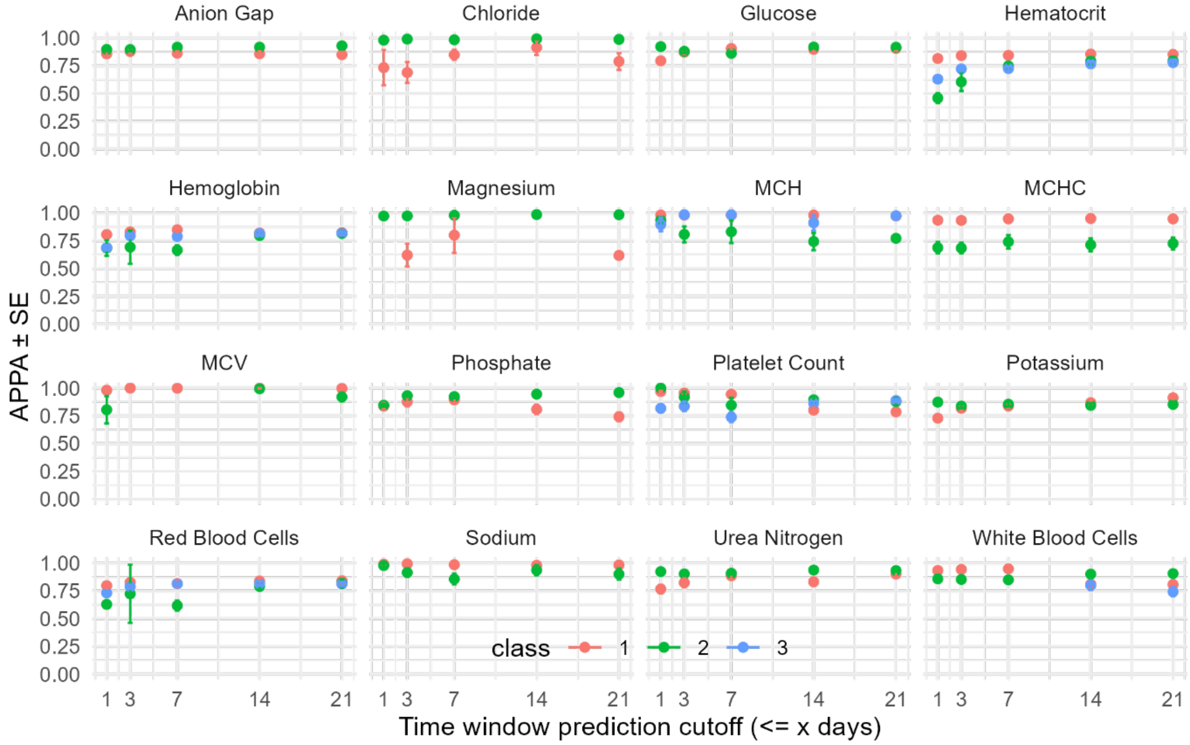

Supplementary Figure 5. Average posterior probability of assignment (APPA) for each trajectory class and marker at each time point window in TRACK SCI. Each point represents the APPA of using the respective model and <= x days of data to predict trajectory membership, where x is the time window cutoff. The standard error of the mean (SE) across PPA for all subjects is represented. Markers with a single trajectory class are not shown as they have an APPA = 1.

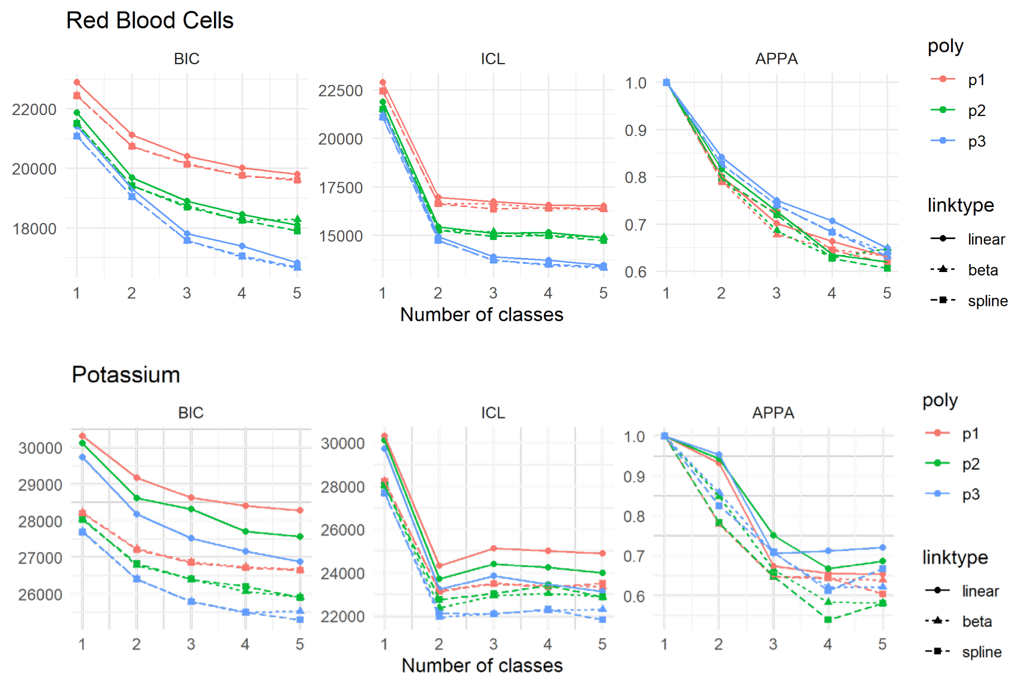

Supplementary Figure 6. Example of model fit plots for two different types of model selection "patterns". The top row shows the BIC, ICL and APPA (mean APPA across classes) for Red Blood Cells. It can be observed that polynomial degree has a higher effect on BIC and ICL than the type of link function. For Potassium (bottom row), the effect of polynomial degree and type of link function is compounded. The higher drop in ICL for both markers from 1 to 2 classes suggests that the major gain in model fit happens when more than 1 class is considered, illustrating the need for modeling heterogeneous populations. BIC: Bayes Information Criterion; ICL: Integrated Complete-Data Likelihood; APPA: Average Posterior Probability of Assignment.

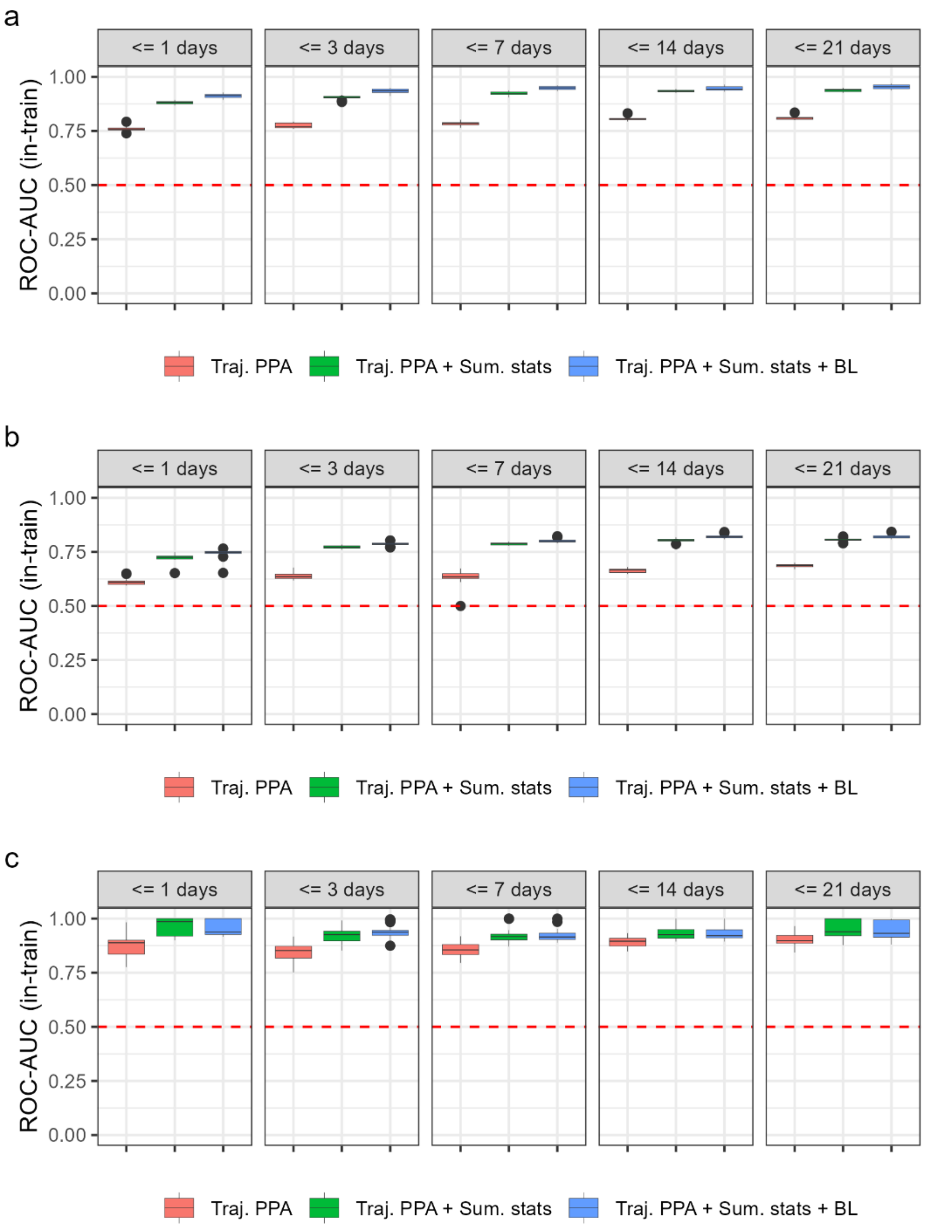

Supplementary Figure 7. ROC-AUC performance of dynamic predictions on the train data. (a) ROC-AUC in-train sample performance of task I in-hospital mortality. (b) ROC-AUC in-train sample performance of task II for detecting the presence of SCI after spine trauma. (c) ROC-AUC in-train sample performance of task III on detecting SCI severity on the TRACK-SCI cohort, external to trajectory modeling. Dashed red lines represent the mean prevalence of the outcome of interest in each experiment. Three predictors' lists are shown: Traj. PPA = posterior probability of trajectory classification only; + Sum. stats = addition of summary statistics of blood data; and + BL = addition of baseline predictors.

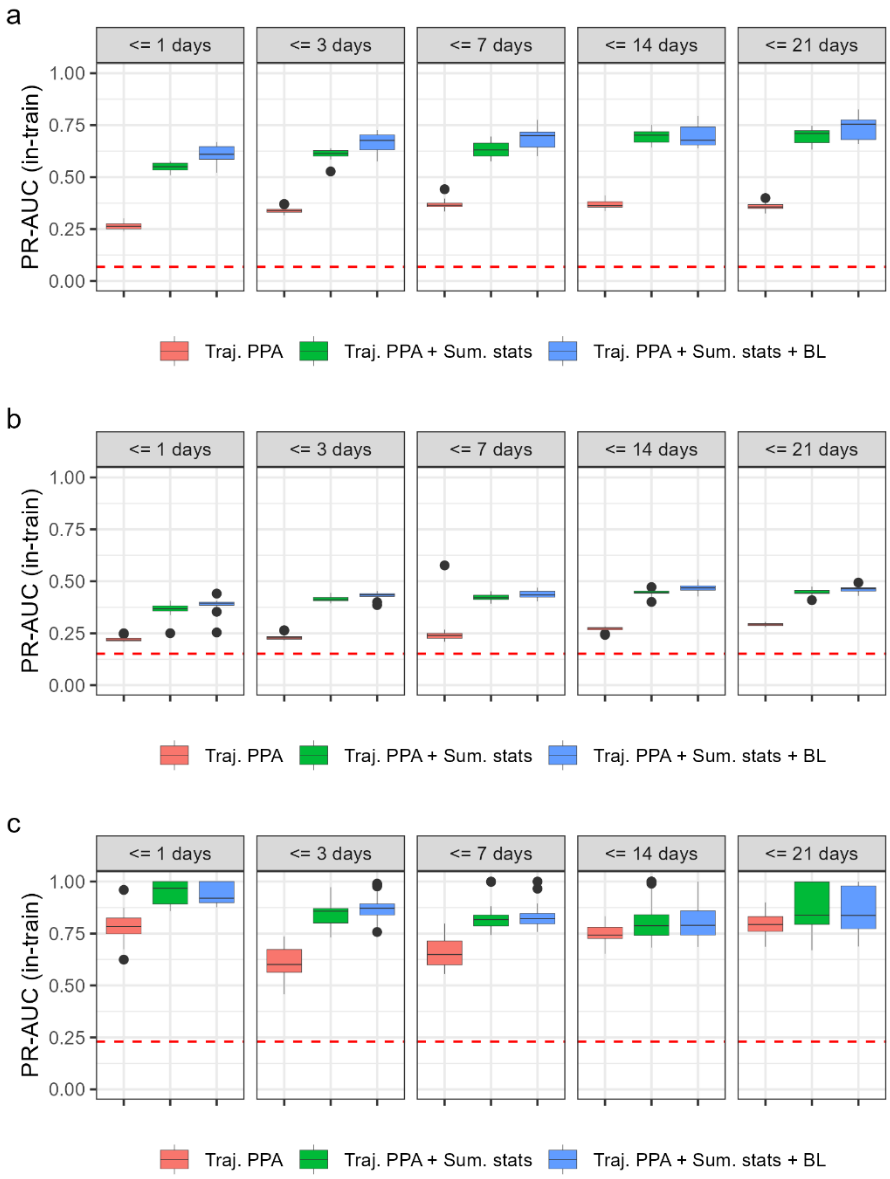

Supplementary Figure 8. PR-AUC performance of dynamic predictions on the train data. (a) PR-AUC in-train sample performance of experiment I in-hospital mortality. (b) PR-AUC in-train sample performance of experiment II for detecting the presence of SCI after spine trauma. (c) PR-AUC in -train sample performance of experiment III on detecting SCI severity on the TRACK-SCI cohort, external to trajectory modeling. Dashed red lines represent the non-information rate (mean prevalence of the outcome of interest in each experiment). Three predictors' lists are shown: Traj. PPA = posterior probability of class assignment only; + Sum. stats = addition of summary statistics of blood data; and + BL = addition of baseline predictors.

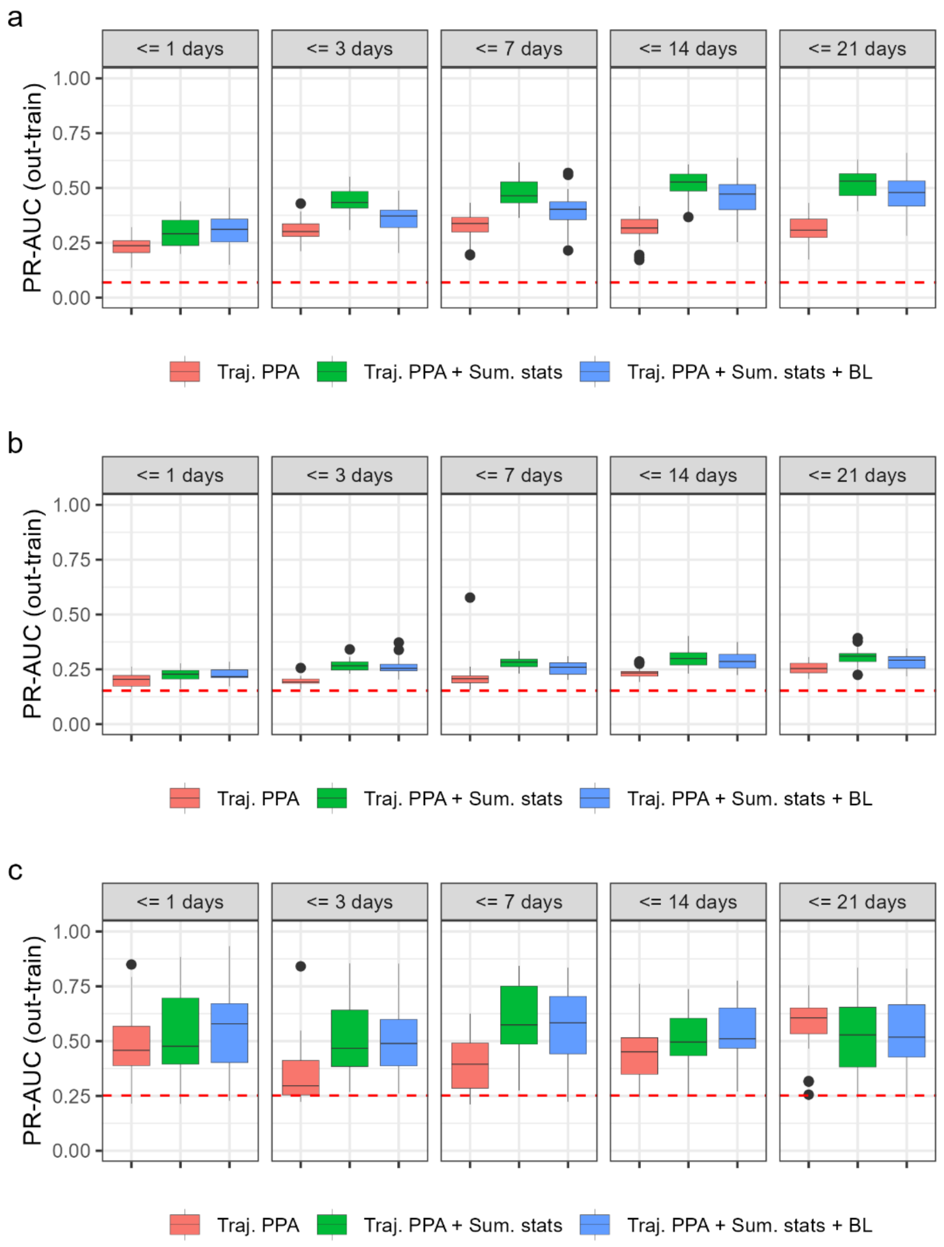

Supplementary Figure 9. PR-AUC performance of dynamic predictions on the out-of-train data. (a) PR-AUC in-train sample performance of experiment I in-hospital mortality. (b) PR-AUC in-train sample performance of experiment II for detecting the presence of SCI after spine trauma. (c) PR-AUC out-of-train sample performance of experiment III on detecting SCI severity on the TRACK-SCI cohort, external to trajectory modeling. Dashed red lines represent the non-information rate (mean prevalence of the outcome of interest in each experiment). Three predictors' lists are shown: Traj. PPA = posterior probability of class assignment only; + Sum. stats = addition of summary statistics of blood data; and + BL = addition of baseline predictors.

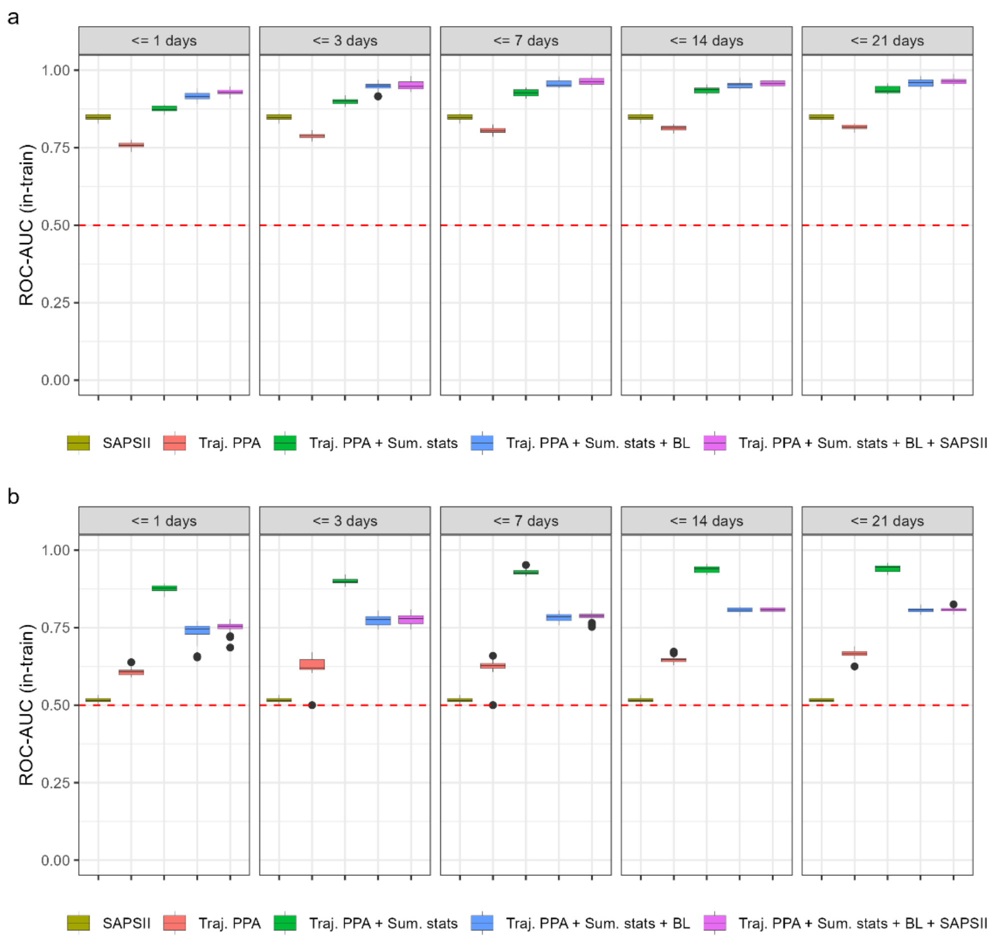

Supplementary Figure 10. ROC-AUC performance of dynamic predictions on the train data for ICU patients with SAPS II. (a) ROC-AUC in-train sample performance of task I in-hospital mortality. (b) ROC-AUC in-train sample performance of task II for detecting the presence of SCI after spine trauma. Dashed red lines represent the mean prevalence of the outcome of interest in each experiment. Four predictors' lists are shown: SAPS = SAPS II score; Traj. PPA = posterior probability of trajectory classification only; + Sum. stats = addition of summary statistics of blood data; and + BL = addition of baseline predictors.

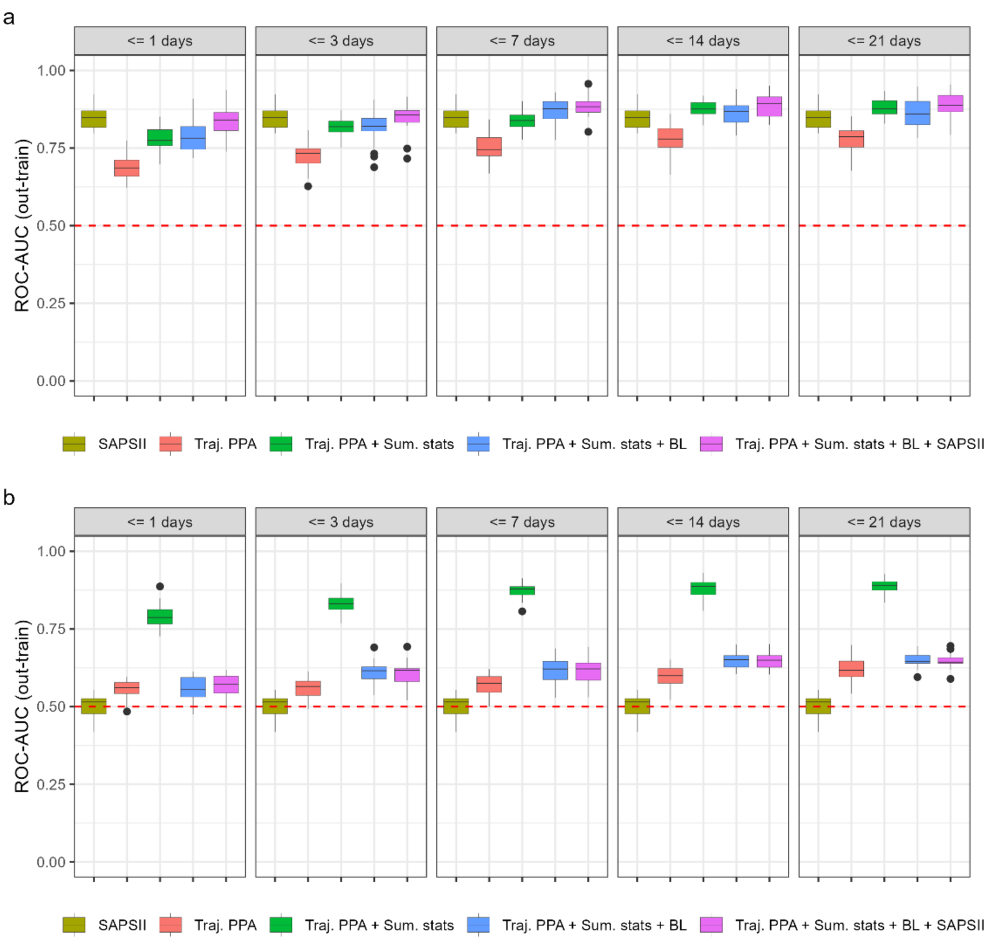

Supplementary Figure 11. ROC-AUC performance of dynamic predictions on the out-of-train data for ICU patients with SAPS II. (a) ROC-AUC out-train sample performance of task I in-hospital mortality. (b) ROC-AUC out-train sample performance of task II for detecting the presence of SCI after spine trauma. Dashed red lines represent the mean prevalence of the outcome of interest in each experiment. Four predictors' lists are shown: SAPS = SAPS II score; Traj. PPA = posterior probability of trajectory classification only; + Sum. stats = addition of summary statistics of blood data; and + BL = addition of baseline predictors.

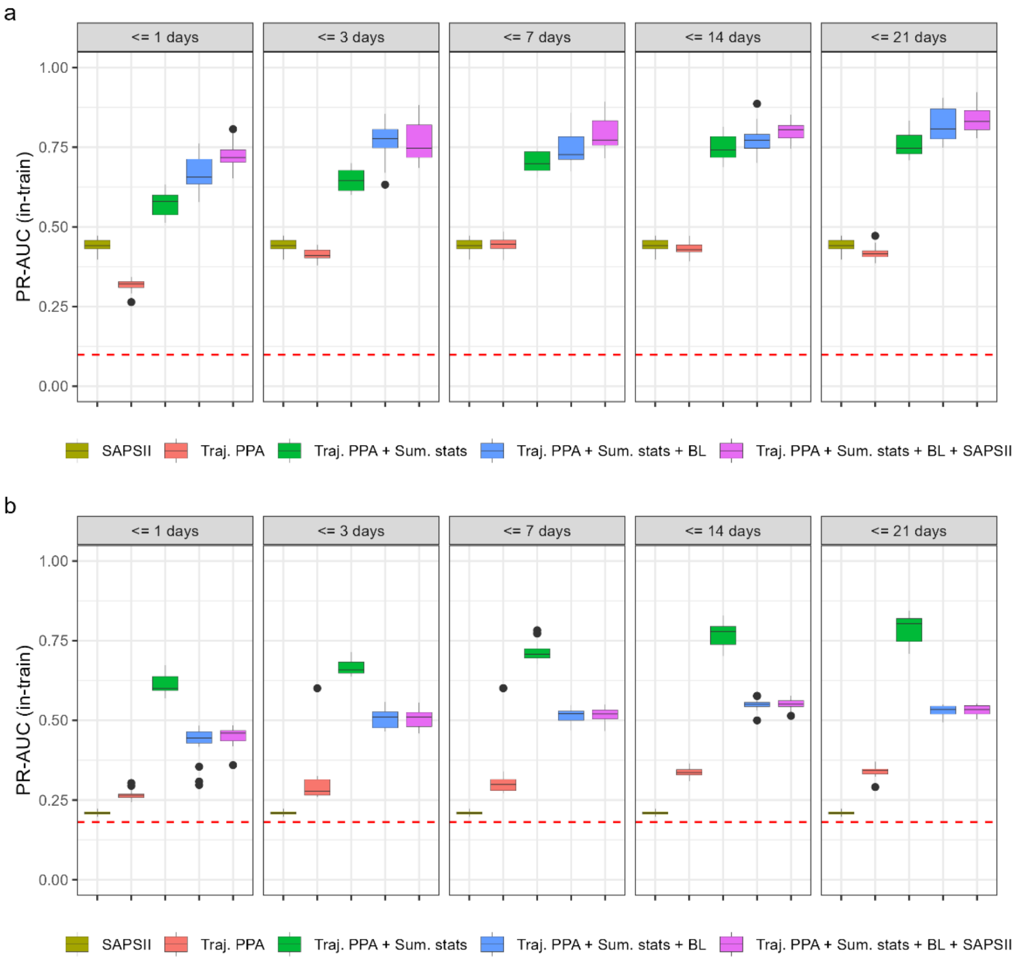

Supplementary Figure 12. PR-AUC performance of dynamic predictions on the train data for ICU patients with SAPS II. (a) PR-AUC in-train sample performance of task I in-hospital mortality. (b) PR-AUC in-train sample performance of task II for detecting the presence of SCI after spine trauma. Dashed red lines represent the mean prevalence of the outcome of interest in each experiment. Four predictors' lists are shown: SAPS = SAPS II score; Traj. PPA = posterior probability of trajectory classification only; + Sum. stats = addition of summary statistics of blood data; and + BL = addition of baseline predictors.

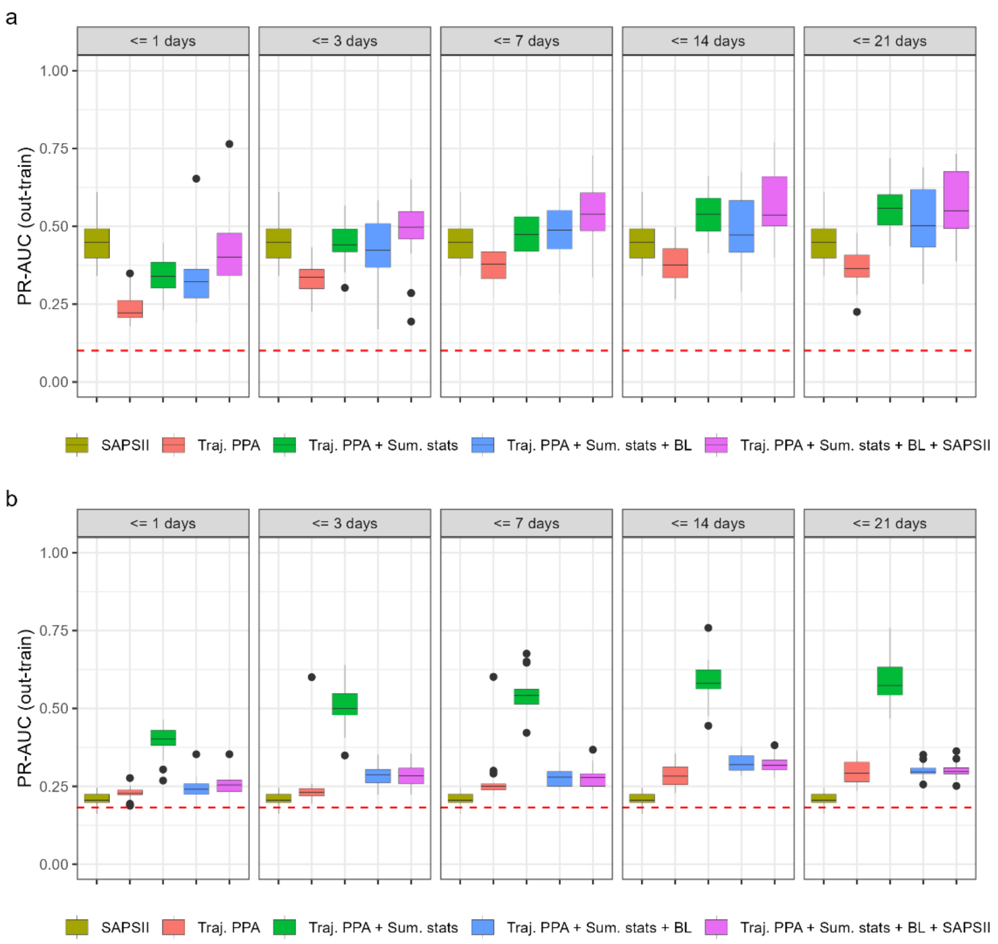

Supplementary Figure 13. PR-AUC performance of dynamic predictions on the out-train data for ICU patients with SAPS II. (a) PR-AUC out-train sample performance of task I in-hospital mortality. (b) PR-AUC out-train sample performance of task II for detecting the presence of SCI after spine trauma. Dashed red lines represent the mean prevalence of the outcome of interest in each experiment. Four predictors' lists are shown: SAPS = SAPS II score; Traj. PPA = posterior probability of trajectory classification only; + Sum. stats = addition of summary statistics of blood data; and + BL = addition of baseline predictors.

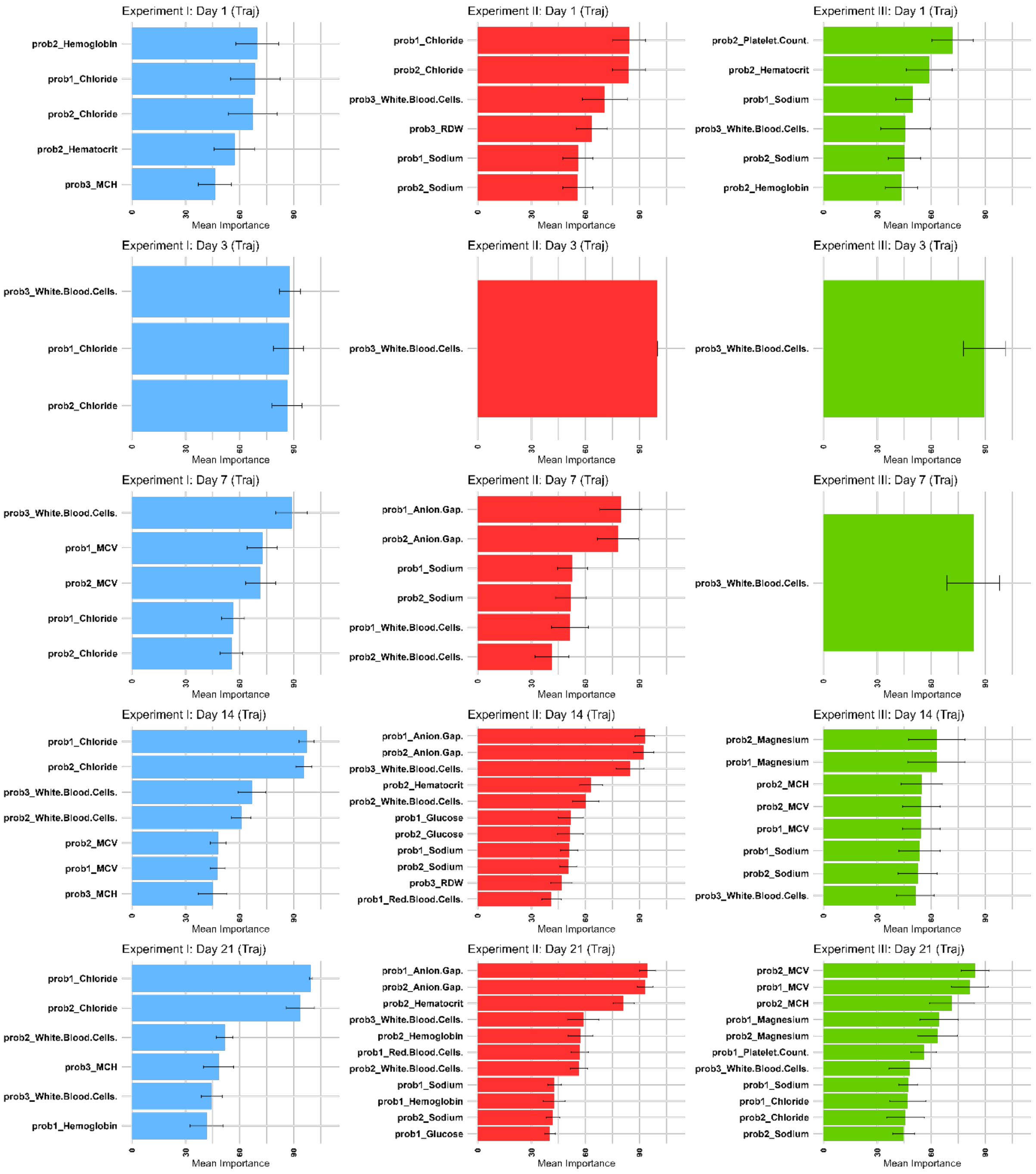

Supplementary Figure 14. Variable importance for models with posterior probability of trajectory classification only. Variables with mean importance of more than 40 for (Left) Experiment I: In-hospital mortality; (Middle) Experiment II: presence of SC; (Right) Experiment III: severity of SCI.

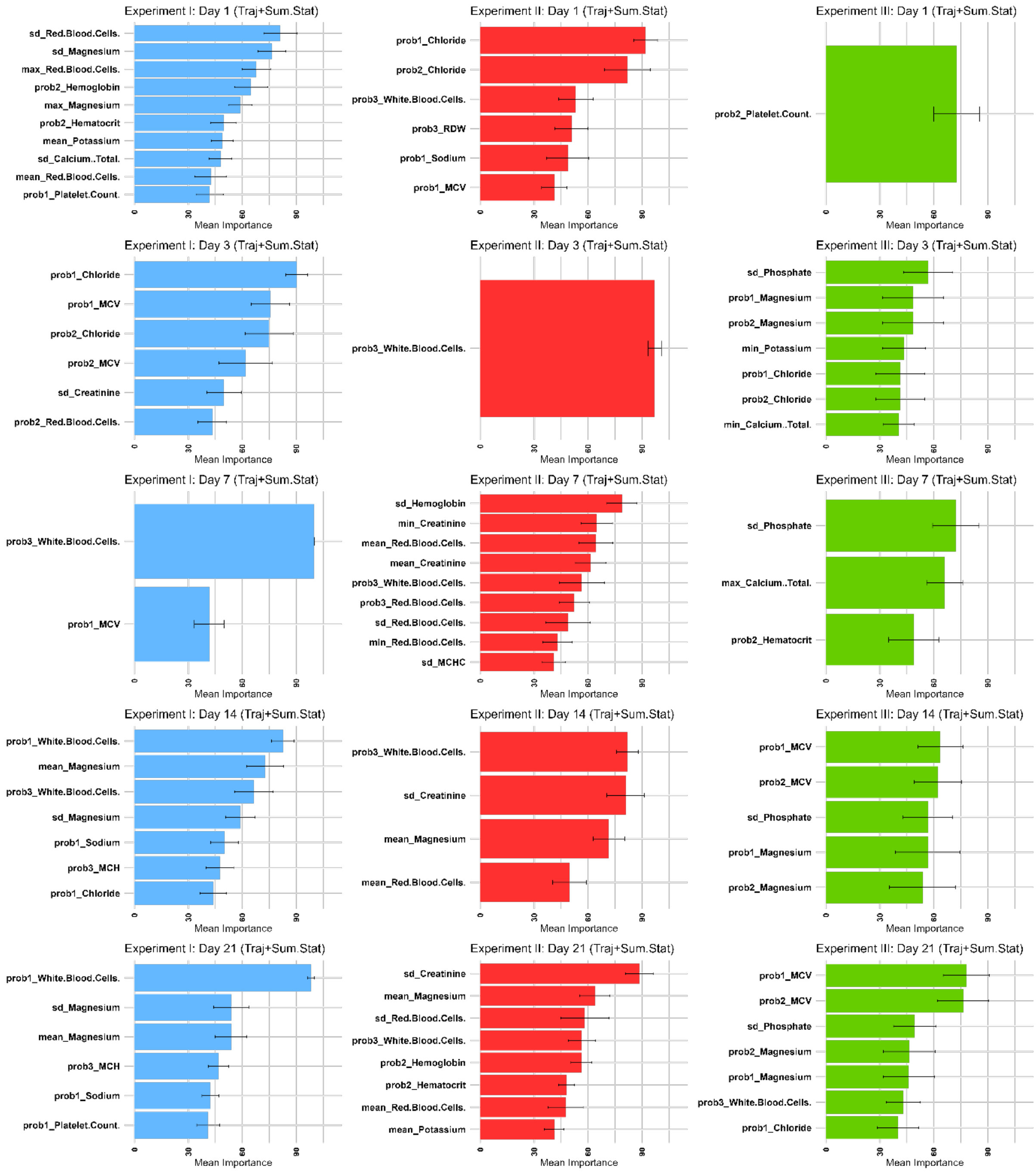

Supplementary Figure 15. Variable importance for models with posterior probability of trajectory classification and summary statistics of biomarkers. Variables with mean importance of more than 40 for (Left) Experiment I: In-hospital mortality; (Middle) Experiment II: presence of SC; (Right) Experiment III: severity of SCI.

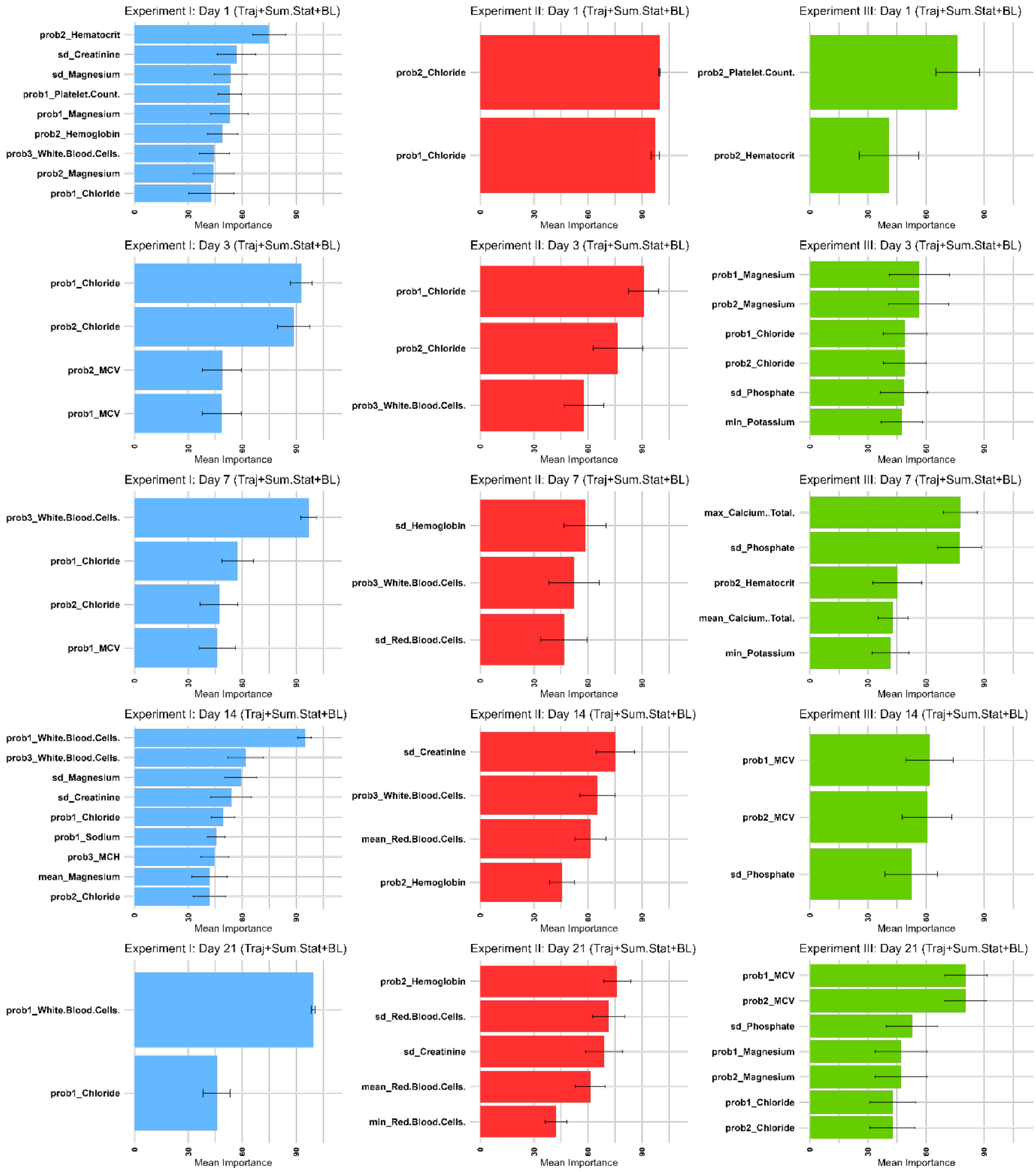

Supplementary Figure 16. Variable importance for models with posterior probability of trajectory classification, summary statistics and baseline predictors. Variables with mean importance of more than 40 for (Left) Experiment I: In-hospital mortality; (Middle) Experiment II: presence of SC; (Right) Experiment III: severity of SCI.
